# Supplementary material for: Erroneous predictive coding across brain hierarchies in a non-human primate model of autism spectrum disorder
Source: Commun Biol. 2024 Jul 12;7:851. doi: 10.1038/s42003-024-06545-3 (PMC11239931; doi:10.1038/s42003-024-06545-3)
Supplement: Supplementary file 2 — Supplementary Information [file 42003_2024_6545_MOESM2_ESM.pdf]

Supplemental Methods

Erroneous Predictive Coding Across Brain Hierarchies in a Non-Human Primate Model of Autism Spectrum Disorder

A Hierarchical Predictive Coding Model for the Local-Global Paradigm

The neural operations in the x stream between Levels S and 1 are shown in Figure S2. Level S contains a neuronal population (denoted by x_s) that receives a sensory input (black arrow) and a prediction signal (green arrow) from Level 1, and sends a prediction-error signal (blue arrow) to Level 1. Level 1 contains a neuronal population (x_1) that receives the prediction-error signal from Level S, and sends a prediction signal to Level S. If we assume that the strengths of the sensory input and the prediction signal are 1 and $P1_x$ ($0 \leq P1_x \leq 1$), respectively, then there are two possible situations: (1) if the last tone is x, then the strength of the prediction-error signal is $1 - P1_x$, (2) if the last tone is not x, then the prediction error is $0 - P1x$ (a negative value) and the strength of the corresponding prediction-error signal is $|0 - P1_x| = P1_x$ ($|\bullet|$ indicates the absolute value). Absolute values are taken because we assume predictions and prediction errors are encoded in neuronal firing rates (1), which can only have non-negative values. Thus, the prediction-error signal received during the last tone at Level 1 in the x stream (denoted as $PE1_x$) is either $1 - P1_x$ or $P1_x$, where the probability of receiving the former is the transition probability from tone x to x (TP_x) and the probability of receiving the latter is $1 - TP_x$ (see the bar graph in Figure S2A).

Figure S2B shows the neural operations in the x stream between Levels 1 and 2. Similar to Level 1, Level 2 contains a neuronal population (x_2) that receives the prediction-error signal from Level 1, and sends a prediction signal $P2_x$ to Level 1. If the sequence is xx, then the prediction-error signal received at Level 1 is $1 - P1_x$ (since Level S receives tone x) and the prediction-error signal received at Level 2 is $|1 - P1_x - P2_x|$. If the sequence is not xx, then the prediction-error signal received at Level 1 is $P1_x$ (since Level S receives not x) and the prediction-error signal received at Level 2 is $|P1_x - P2_x|$. Thus, the prediction-error signal received during the last tone at Level 2 in the x stream (denoted as $PE2_x$) is either $|1 - P1_x -$

$P2_x|$ or $|P1_x - P2_x|$, where the probability of receiving the former is the sequence probability of sequence xx (SP_{xx}) and the probability of receiving the latter is $1 - SP_{xx}$.

Based on the model, the strengths of the prediction signals (e.g. $P1_x$ and $P2_x$ in the x stream) are to minimize the mean-squared error received at that level, and can be determined once the transition and sequence probabilities are known (see Supplemental methods below). Figure S2C shows the complete model for the xx and xy sequences in the xx and xy blocks, which includes the values of prediction and prediction-error signals at both the local and global levels in both the x and y streams. Note that the same prediction signals appear for both the xx and xy sequences, since predictions occur before the last tone arrives. Furthermore, even though the x and y tones are processed in separate streams based on the tonotopic organization, two streams need to integrate information at Levels 1 and 2 to compute transition probabilities (TP_x and TP_y,) and sequence probabilities (SP_{xx} and SP_{xy}), respectively. In Figure S2C, we indicate these integrations for probability computations as horizontal gray bars between populations x_1 and y_1 and between populations x_2 and y_2.

The deviant responses in xy|xx – xx|xx and xy|xy – xx|xy then can be calculated for the x and y streams by subtracting the model values for the xx sequence from those for the xy sequence in each block (Figure S2D). Note that only the prediction-error signals were left in the deviant responses since the same prediction signals are shared between xx and xy sequences. Furthermore, even though x and y streams are modeled separately, their prediction-error values were combined for later model-fitting (Figure S2E). This is based on the assumption that ECoG recordings offer insufficient spatial resolution to separate the x and y streams. As results, the local prediction error (PE1) and global prediction error (PE2) contained the

deviant responses are 1.50 and 0.88, respectively in xy|xx – xx|xx, and are 1.14 and –0.70, respectively in xy|xy – xx|xy.

Supplemental methods: model calculation

In the model, the optimal value of each prediction signal is to minimize the mean-squared error received. For example, the mean squares of $PE1_x$ (denoted by $MSPE1_x$) can be devised as (based on the bar graph in Figure S2A):

$$MSPE1_x = TP_x * ({s_0}^{n-1} - P1_x)^2 + (1 - TP_x) * (P1_x)^2 \quad [1]$$

The minimums occur when:

$$P1_x = {s_0}^{n-1} * TP_x \quad [2]$$

And $P1_y$ can be obtained in the same fashion:

$$P1_y = TP_y \quad [3]$$

This represent the optimal prediction where first level prediction errors are minimized. Then we added the scaling factor s_1 to $P1_x$ and $P1_y$ and calculate the mean squares of $PE2_x$ (denoted by $MSPE2_x$):

$$MSPE2_x = SP_{xx} * (|{s_0}^{n-1} - P1_x * s_1| - P2_x)^2 + (1 - SP_{xx}) * (P1_x * s_1 - P2_x)^2 \quad [4]$$

The minimums occur when:

$$P2_x = SP_{xx} * (|{s_0}^{n-1} - P1_x * s_1|) + (1 - SP_{xx}) * P1_x * s_1 \quad [5]$$

And $P2_y$ can be obtained in the same fashion:

$$P2_y = SP_{xy} * \left(|1 - P1_y * s_1|\right) + \left(1 - SP_{xy}\right) * P1_y * s_1 \quad [6]$$

Note that the $P2_x$ and $P2_y$ here represent the optimal predictions when potential erroneous predictions at the first level are considered. Also, s_2 was applied to calculate the second level prediction errors, i.e. $P2_x*s_2$ and $P2_y*s_2$ were used (as shown in Figure 5B).

Based on the model, all prediction signals are determined once the transition probabilities (TP_x and TP_y), sequence probabilities (SP_{xx} and SP_{xy}), and scaling factors (s_o, s_1, and s_2) are known. The transition probabilities can be calculated based on the number of tones in a sequence and the sequence probabilities (the MATLAB code for these calculations is provided).

Supplemental references

1. A. Pouget, J. M. Beck, W. J. Ma, P. E. Latham, Probabilistic brains: knowns and unknowns. *Nat. Neurosci.* **16**, 1170–1178 (2013).

Supplemental Figure S1

Erroneous Predictive Coding Across Brain Hierarchies in a Non-Human Primate Model of Autism Spectrum Disorder

Figure S1. All ICs and Deviant Responses. All ICs for each subject: Ji (96 ICs), Rc (92 ICs), Yo (88 ICs), Ca (90 ICs), and Rm (84 ICs), alongside their respective deviant responses. The following information is illustrated in separate panels for each IC, organized vertically: (1) Spatial weights. (2) Time courses across all trials, with five vertical lines indicating the tones' onset. (3) Mean and standard error of the mean (SEM) for all trials. The mean is depicted by a blue line, with the SEM in red. A consistent y-axis scale is maintained for all ICs within each subject. SEM is typically small and may not be easily visible, except for certain cases such as ICs 1 to 5 in Rm. (4) Deviant responses for contrast xy|xx – xx|xx, with significant deviation responses outlined in black. (5) Deviant responses for contrast xy|xy – xx|xy. The bottom two panels sharing a common colorbar for all ICs per subject.

Subject: Ji (1/7)

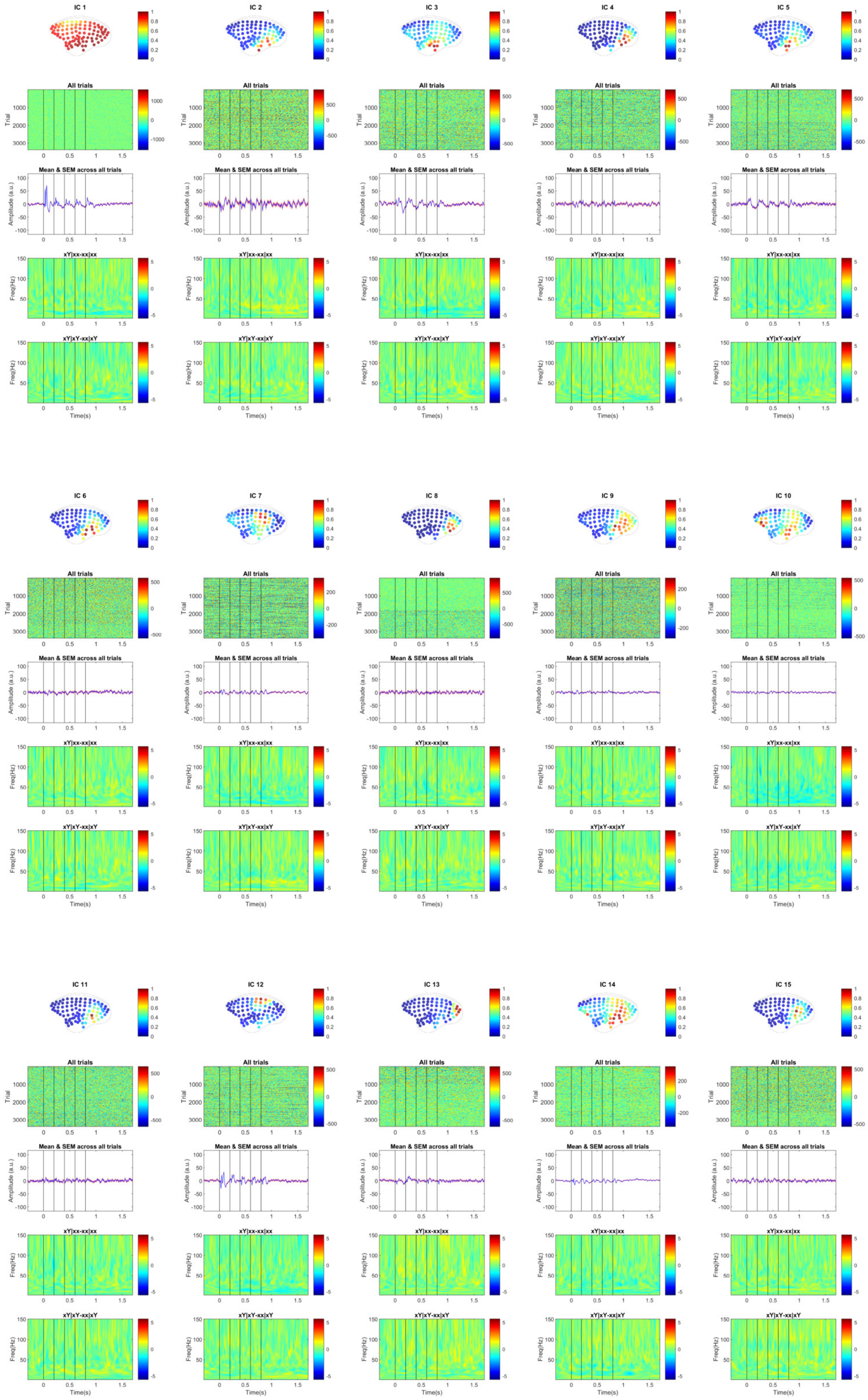

Subject: Ji (2/7)

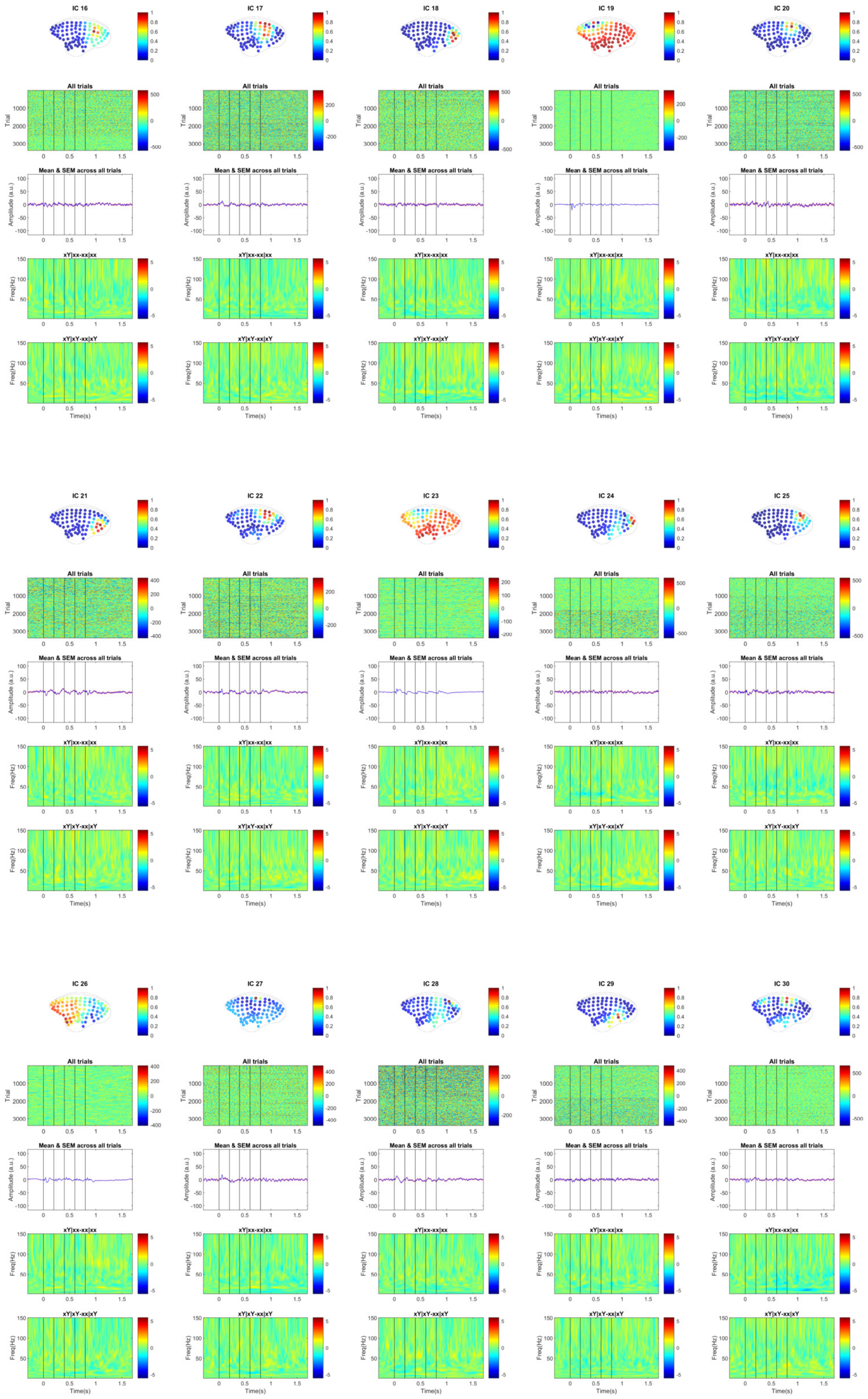

Subject: Ji (3/7)

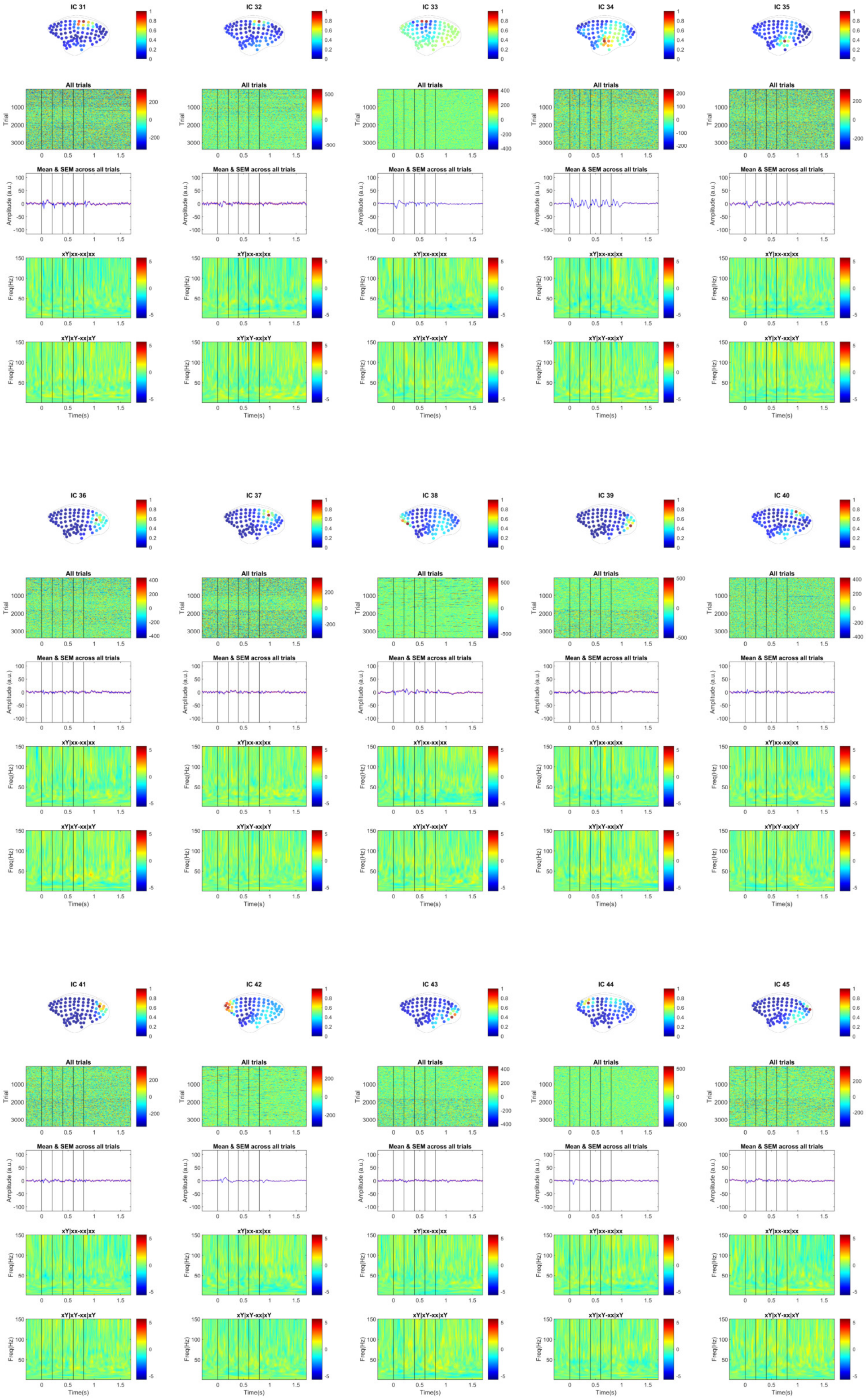

Subject: Ji (4/7)

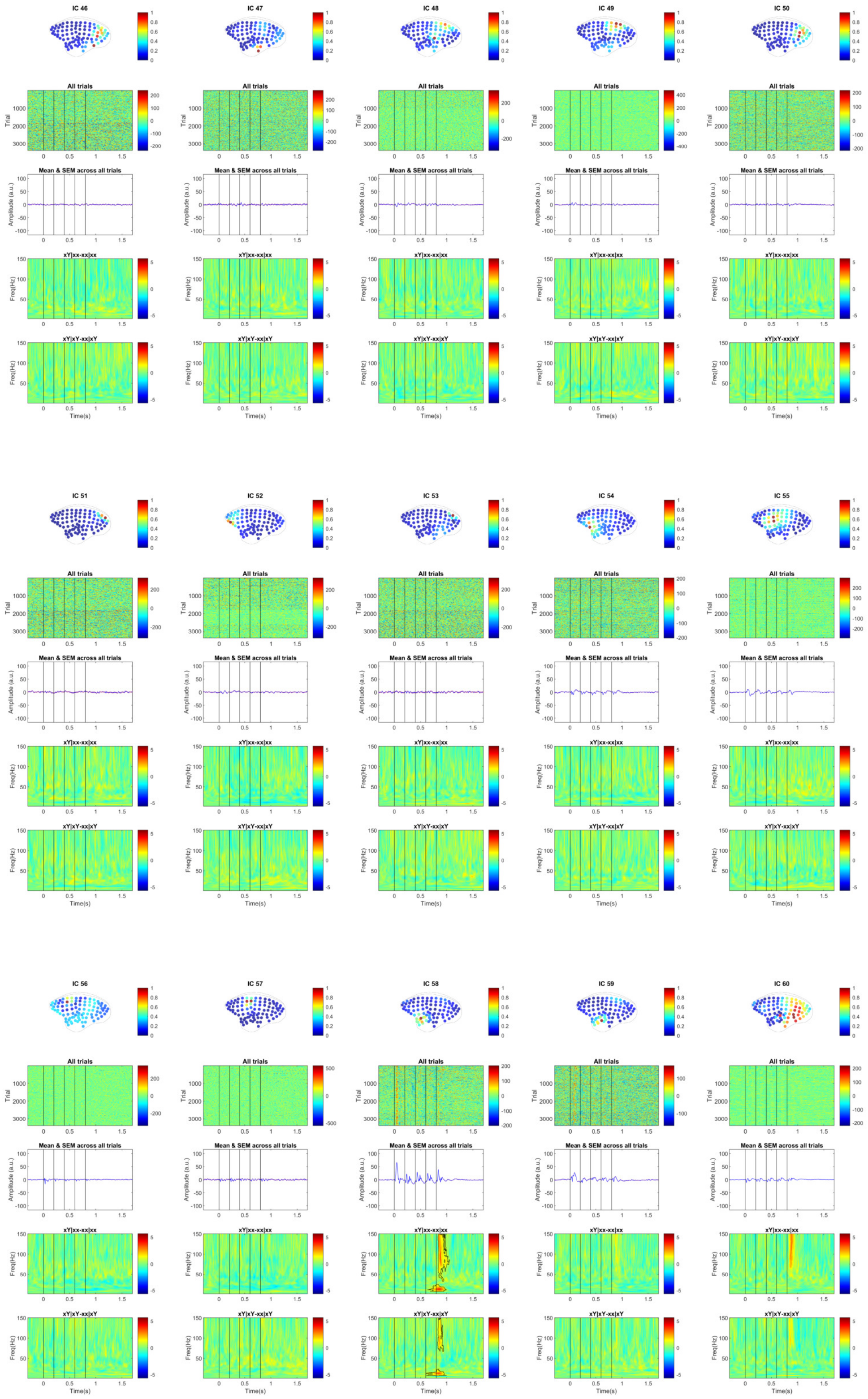

Subject: Ji (5/7)

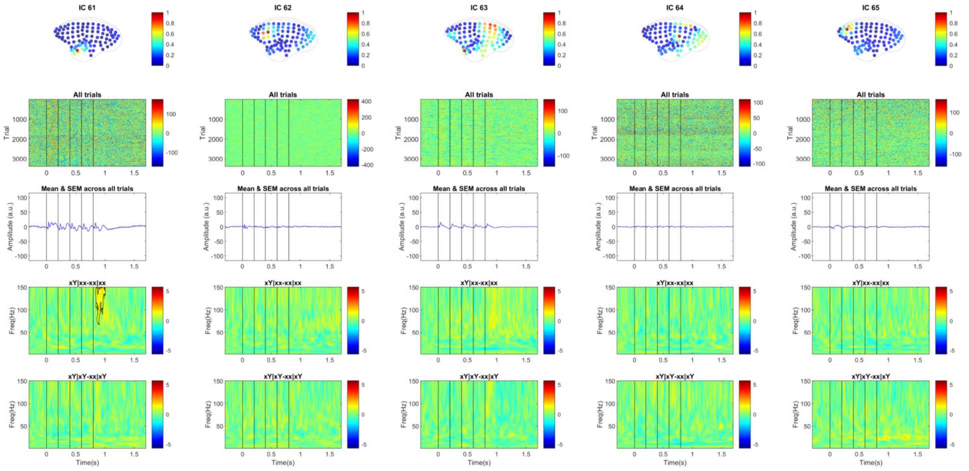

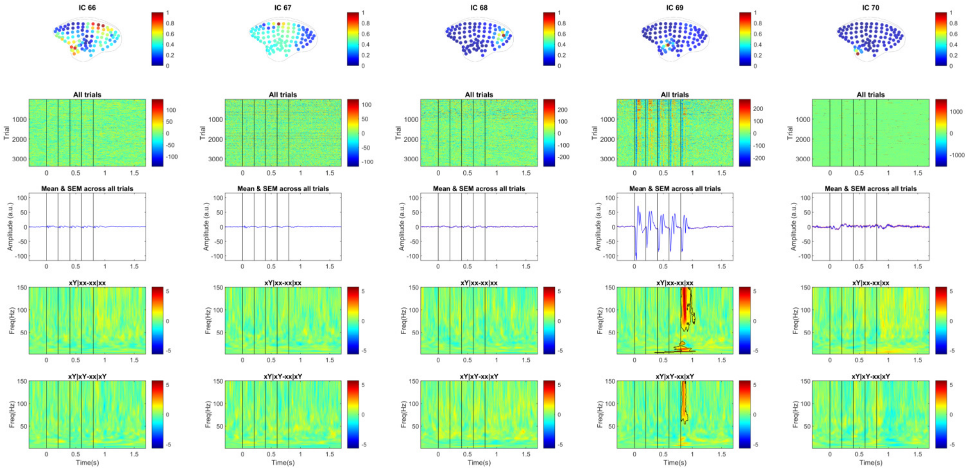

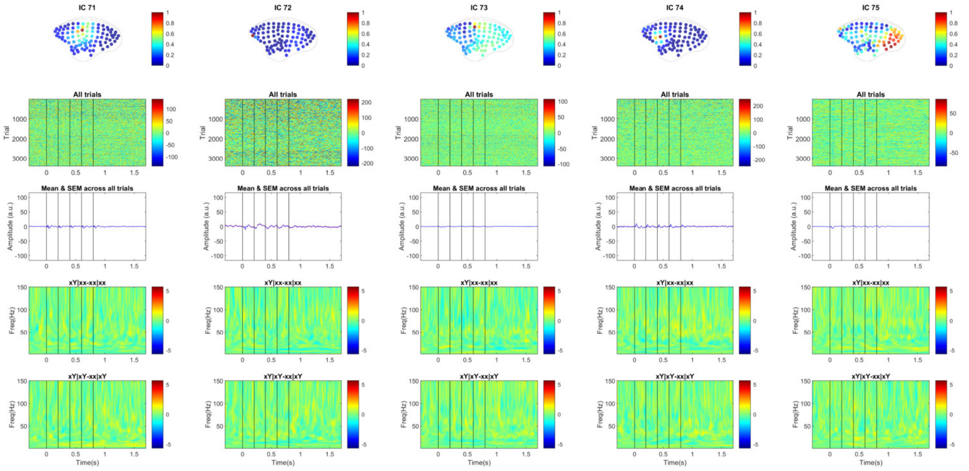

Subject: Ji (6/7)

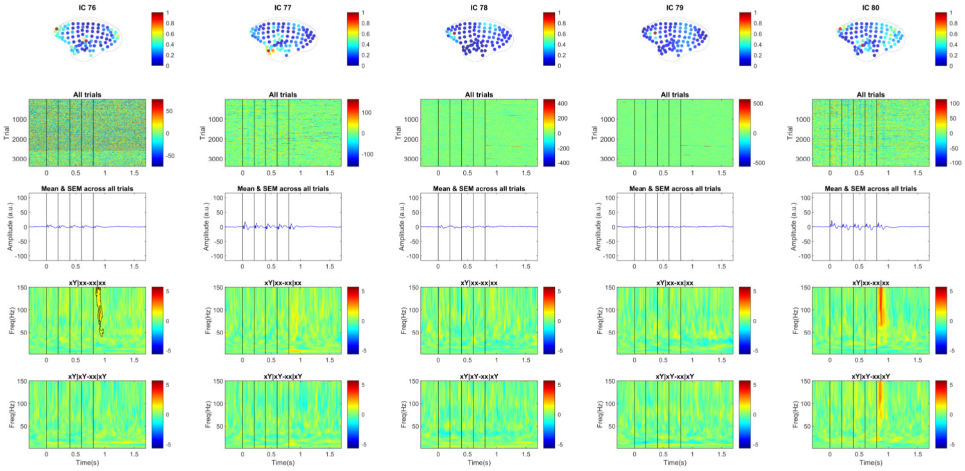

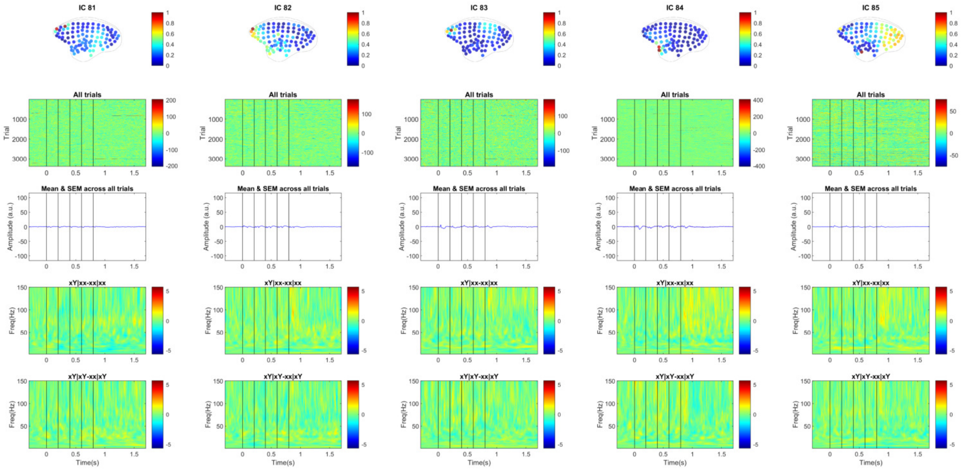

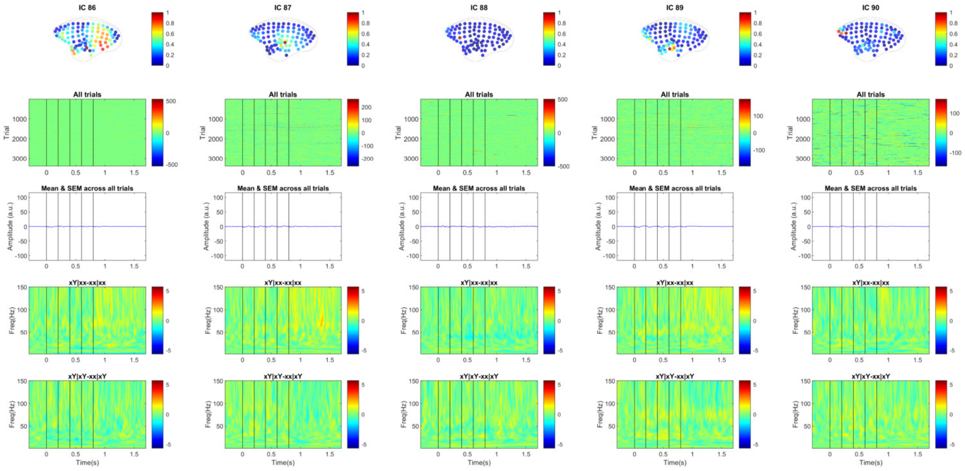

Subject: Ji (7/7)

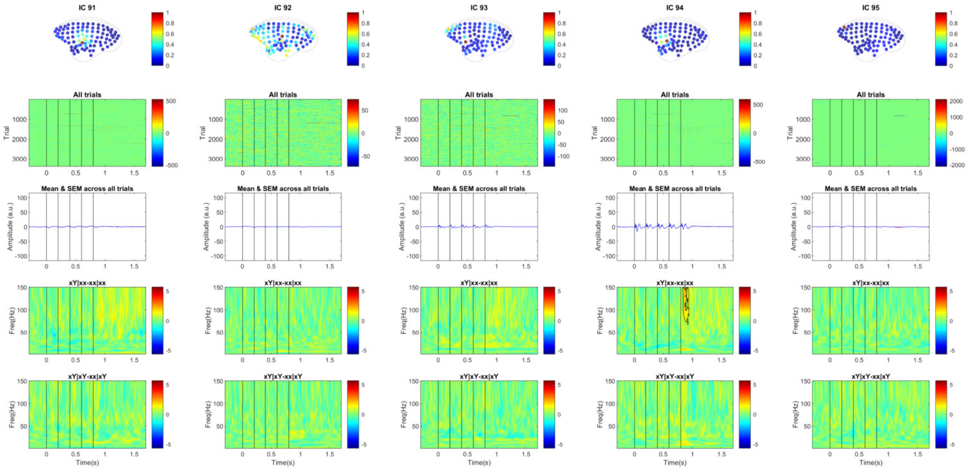

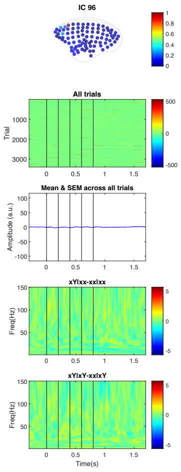

Subject: Rc (1/7)

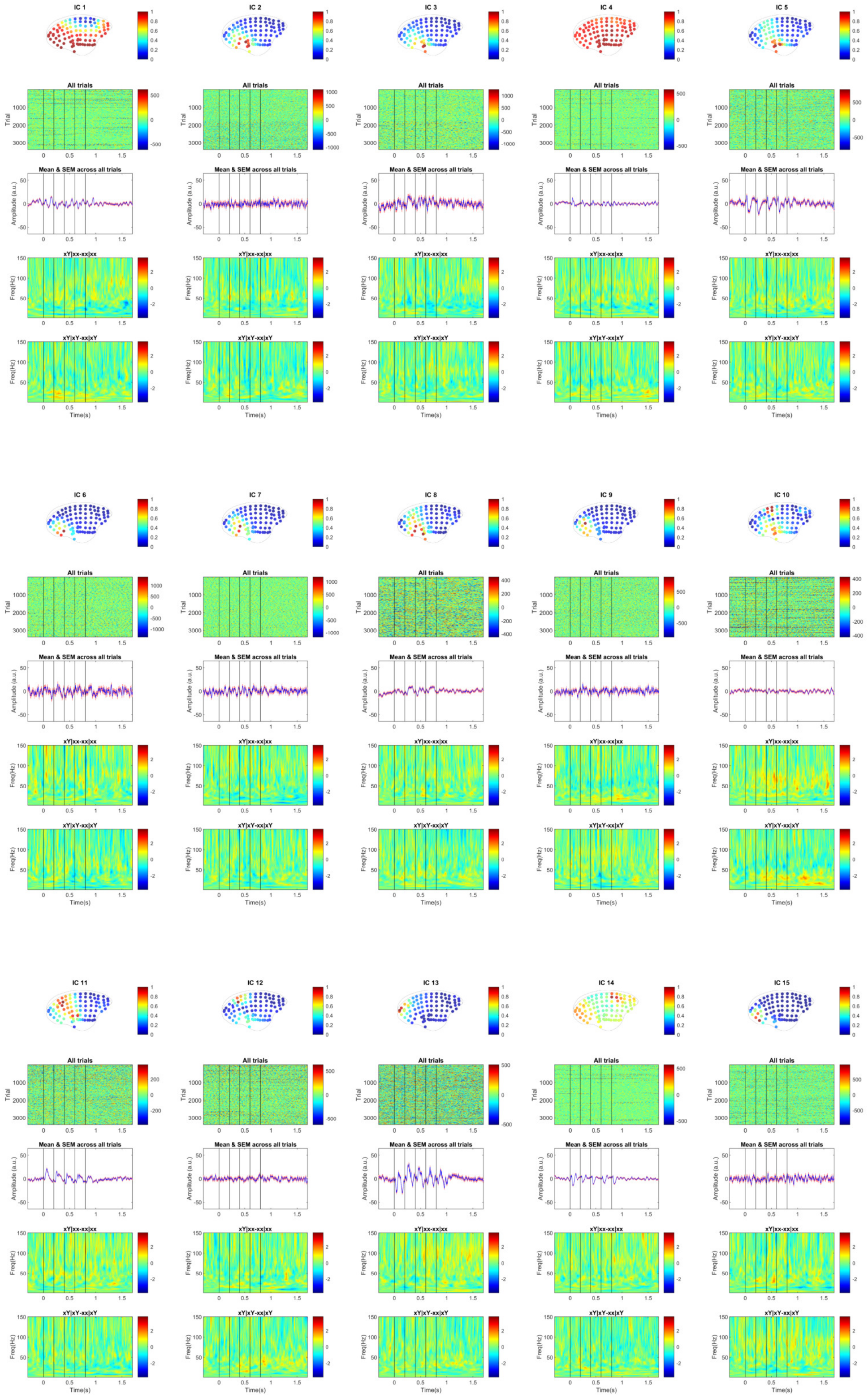

Subject: Rc (2/7)

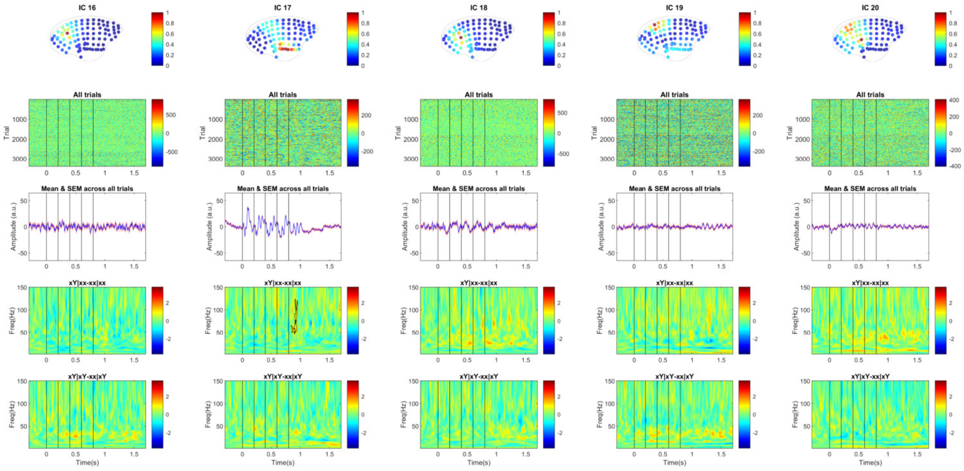

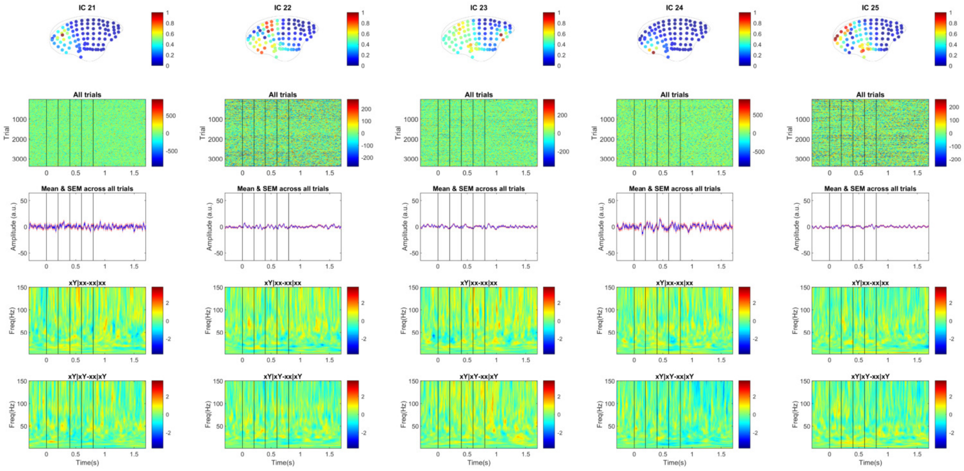

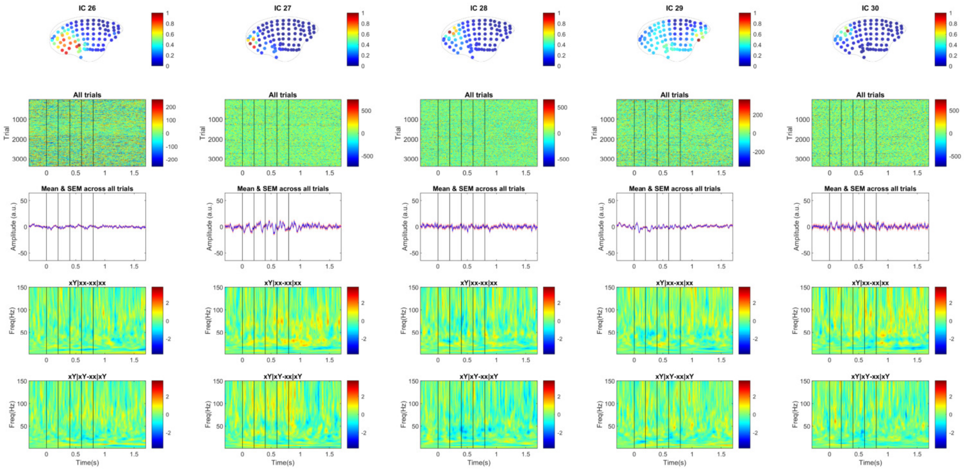

Subject: Rc (3/7)

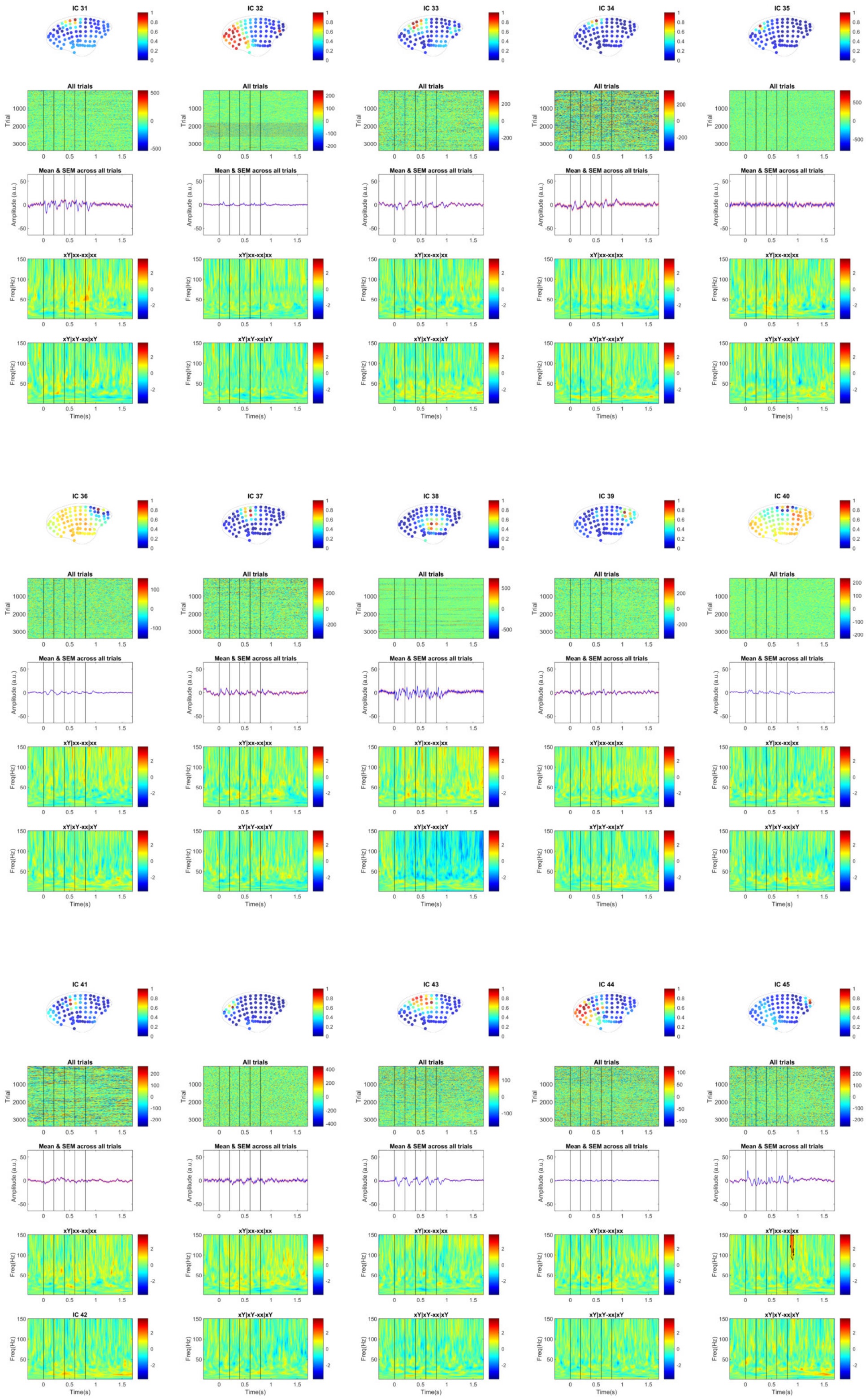

Subject: Rc (4/7)

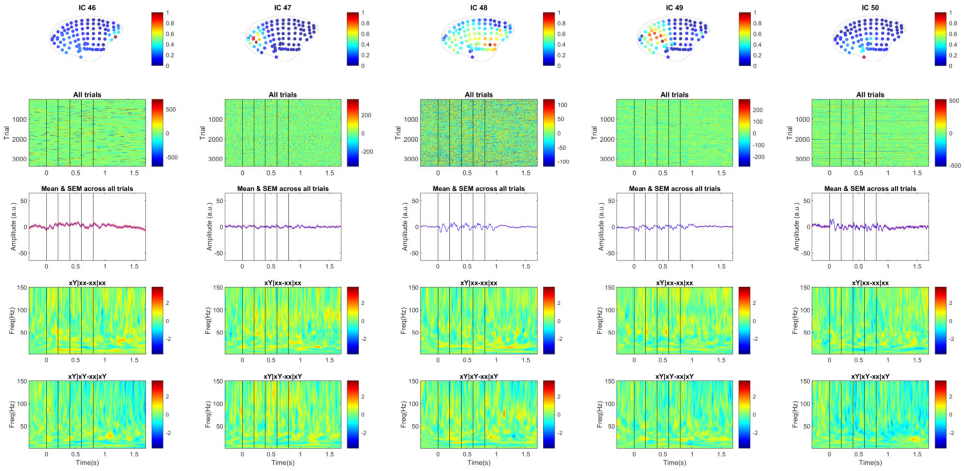

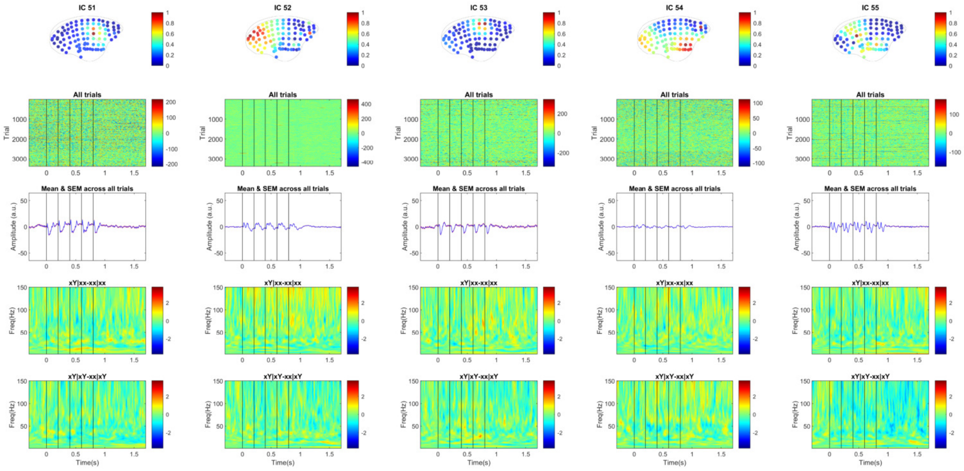

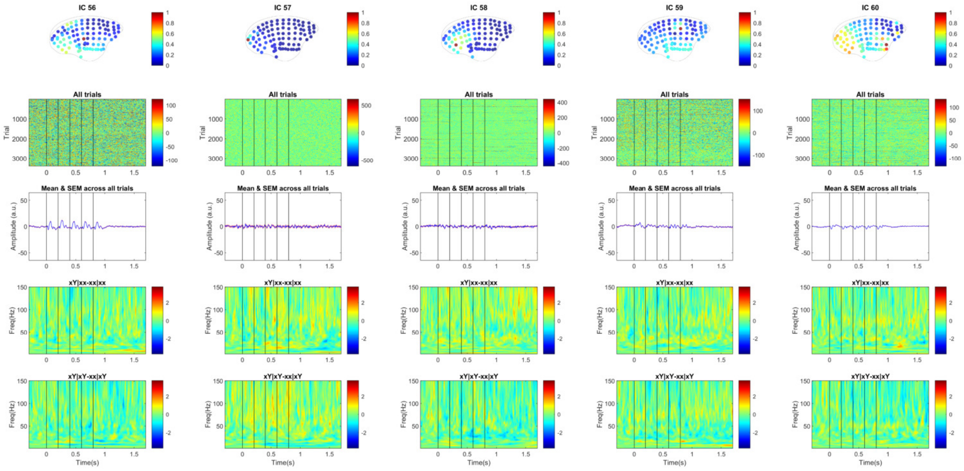

Subject: Rc (5/7)

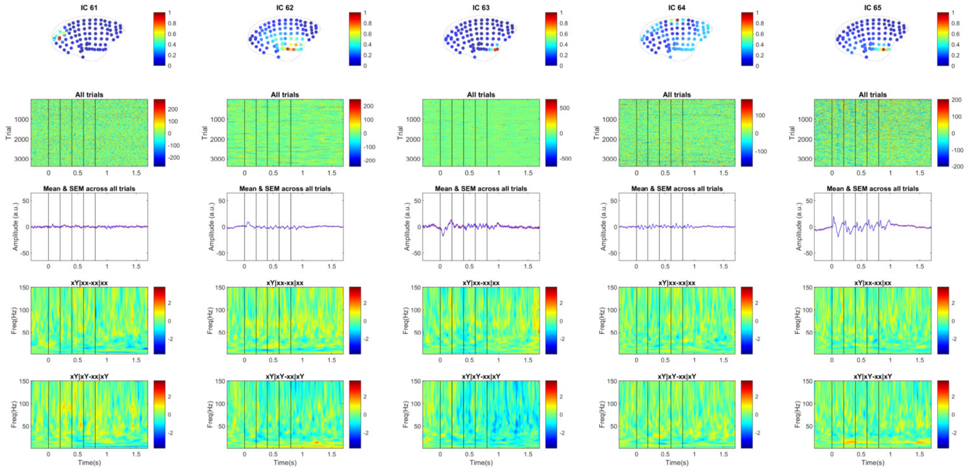

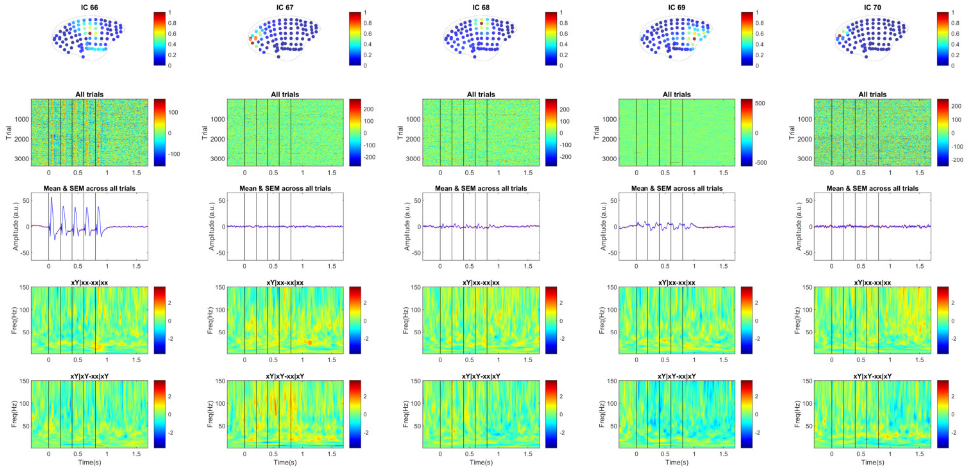

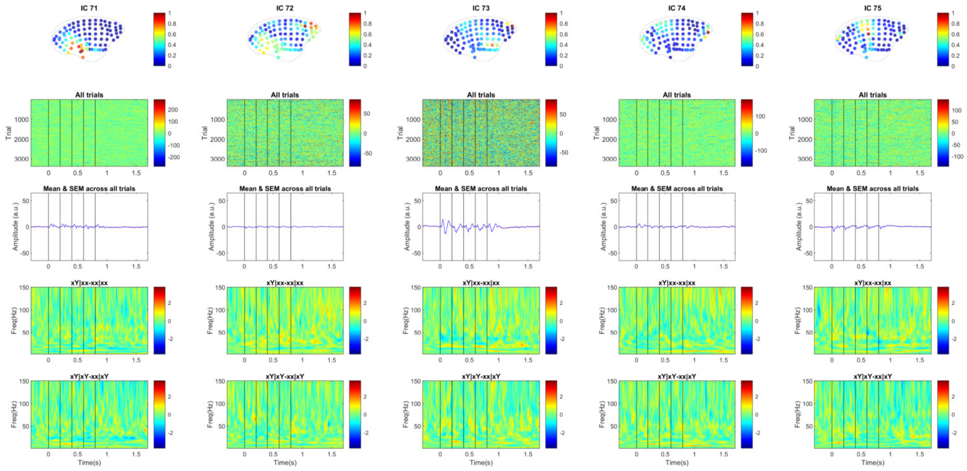

Subject: Rc (6/7)

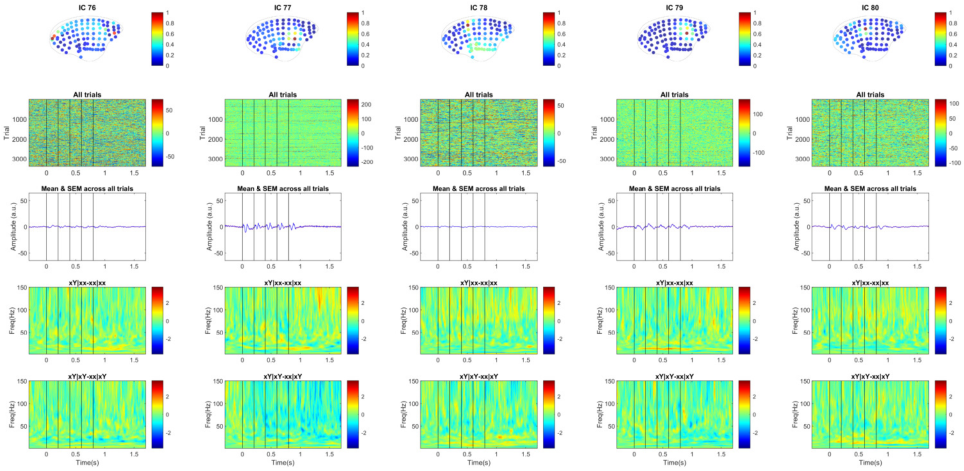

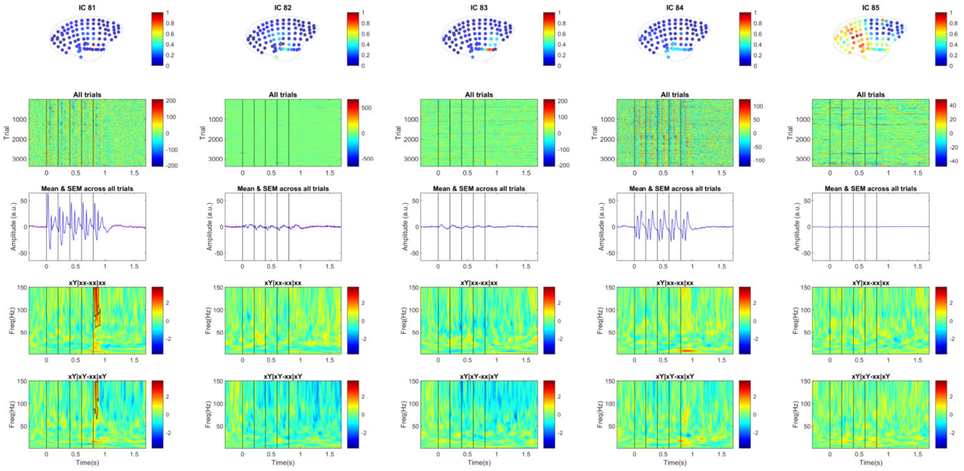

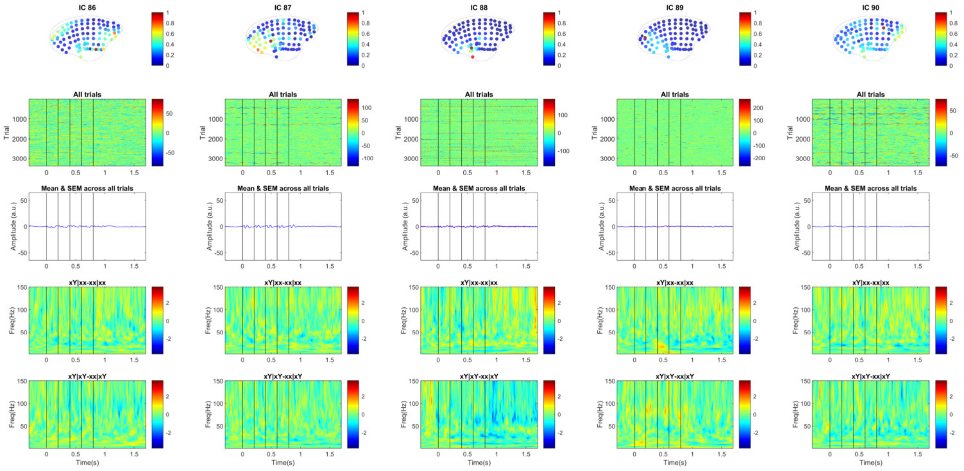

Subject: Rc (7/7)

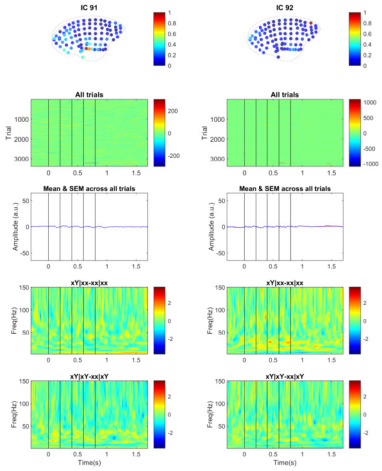

Subject: Yo (1/6)

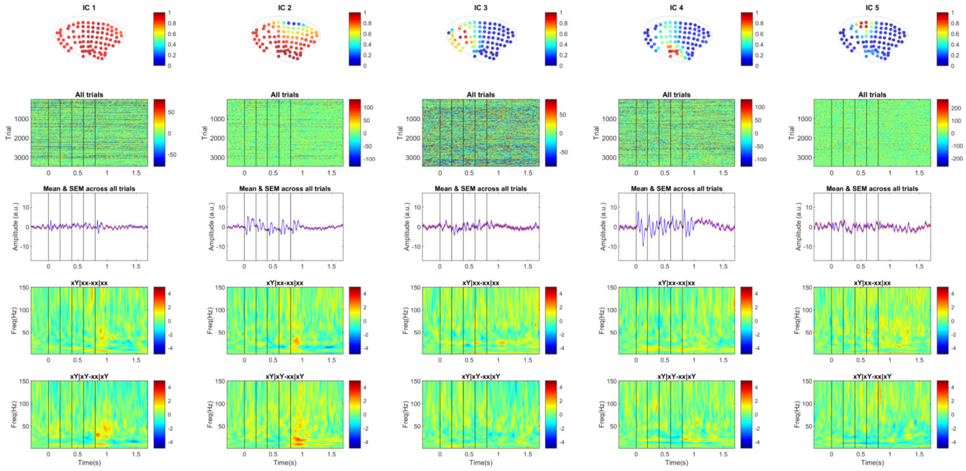

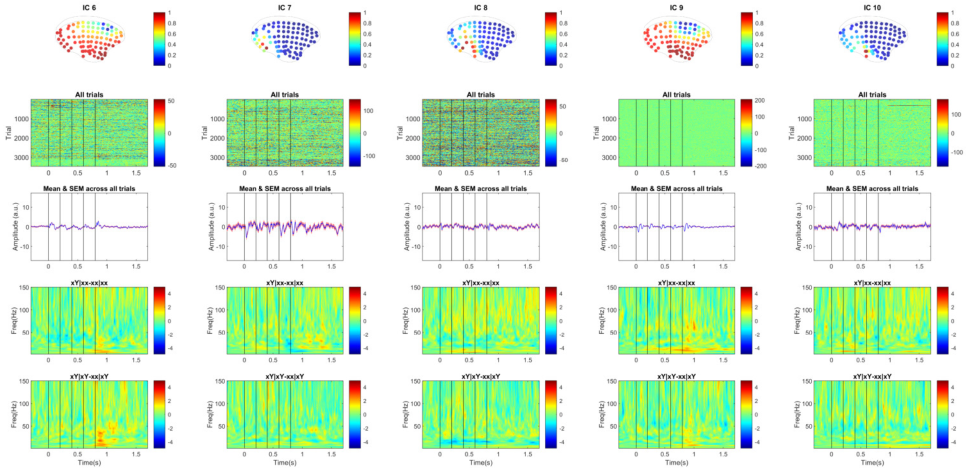

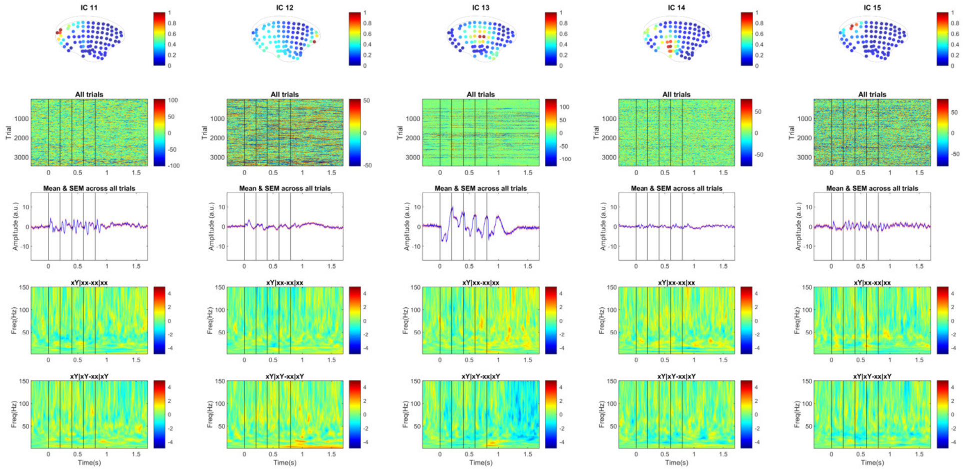

Subject: Yo (2/6)

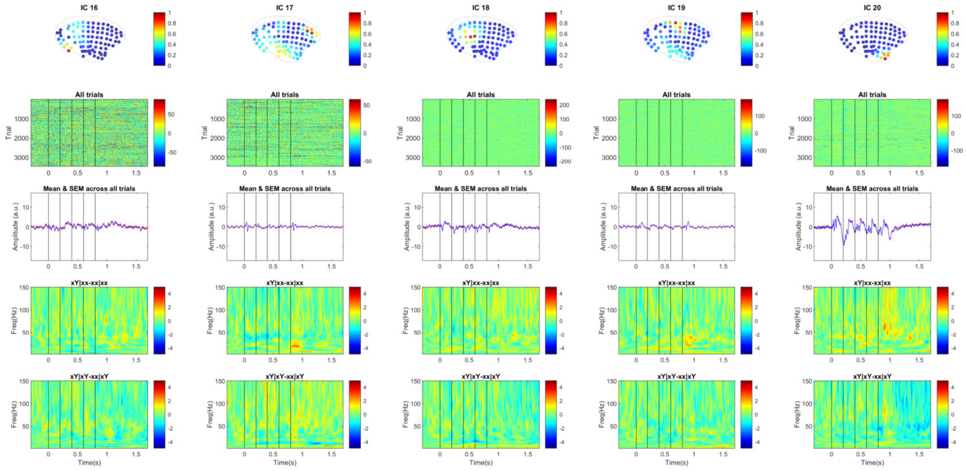

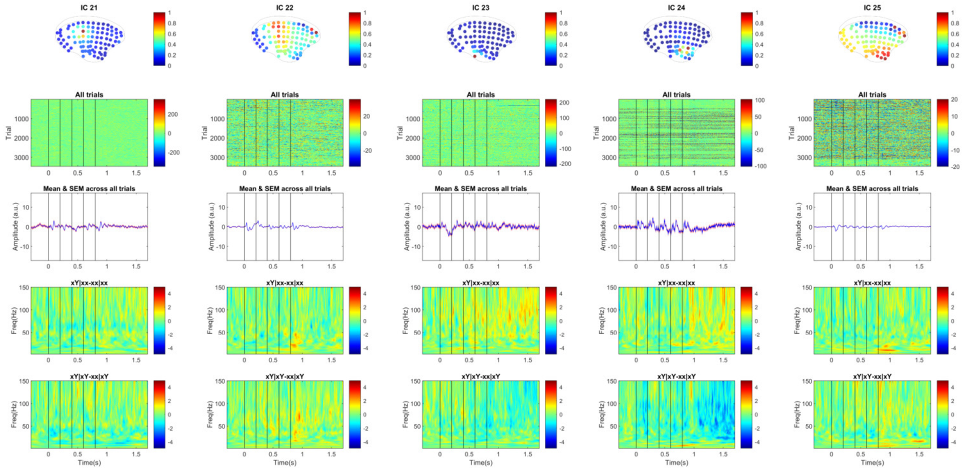

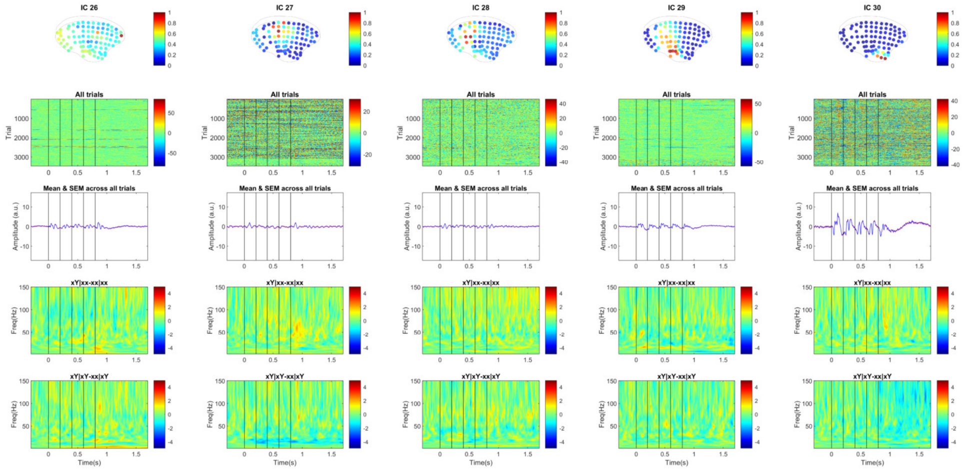

Subject: Yo (3/6)

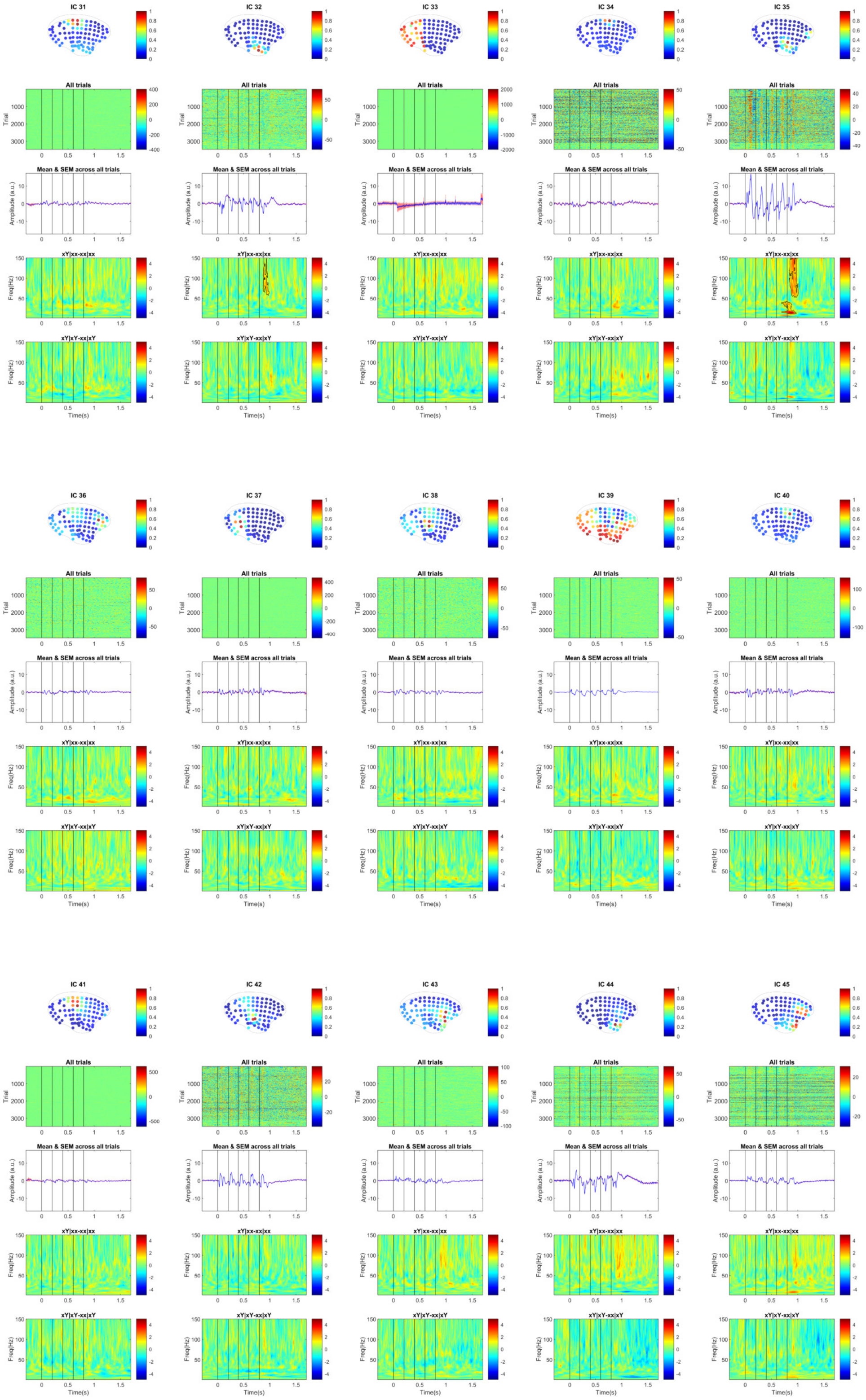

Subject: Yo (4/6)

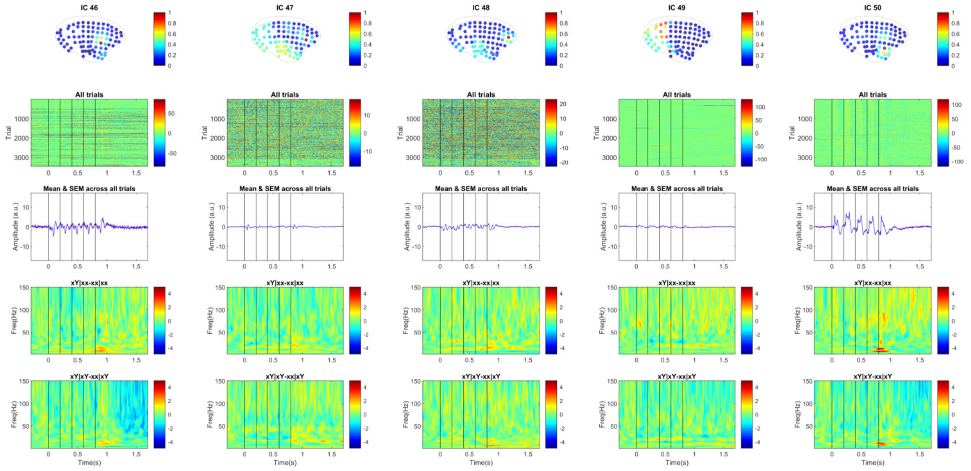

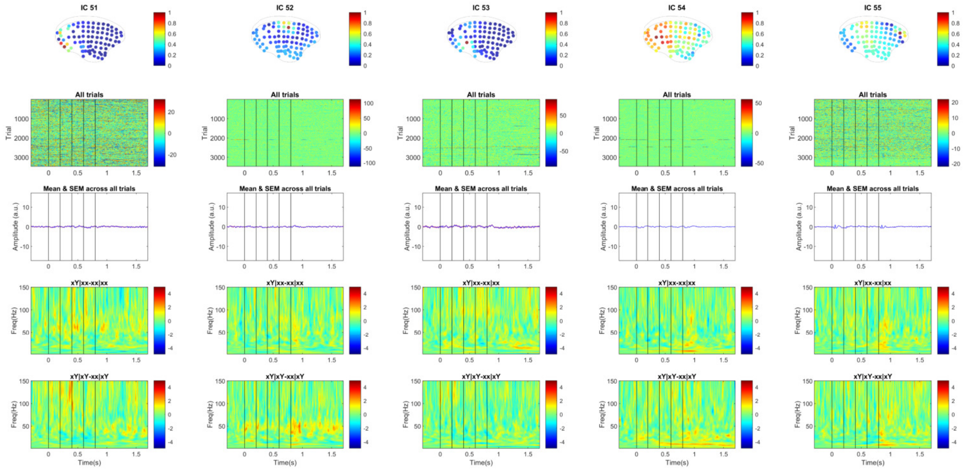

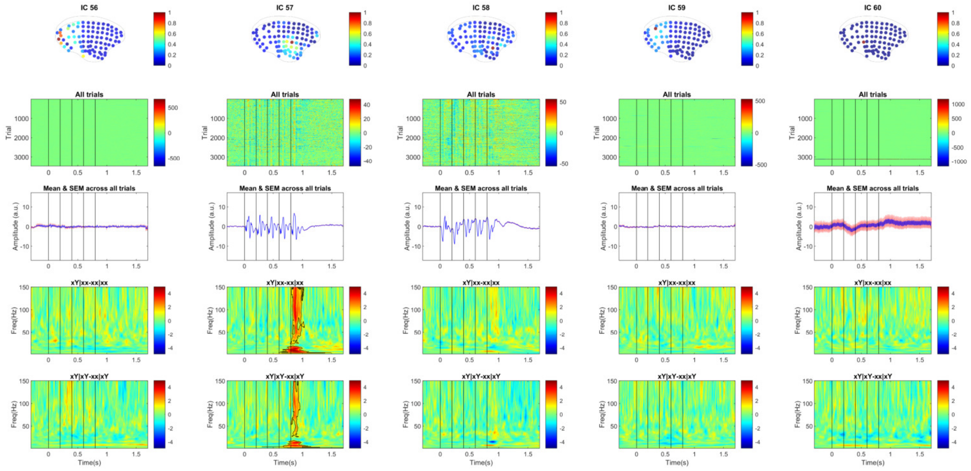

Subject: Yo (5/6)

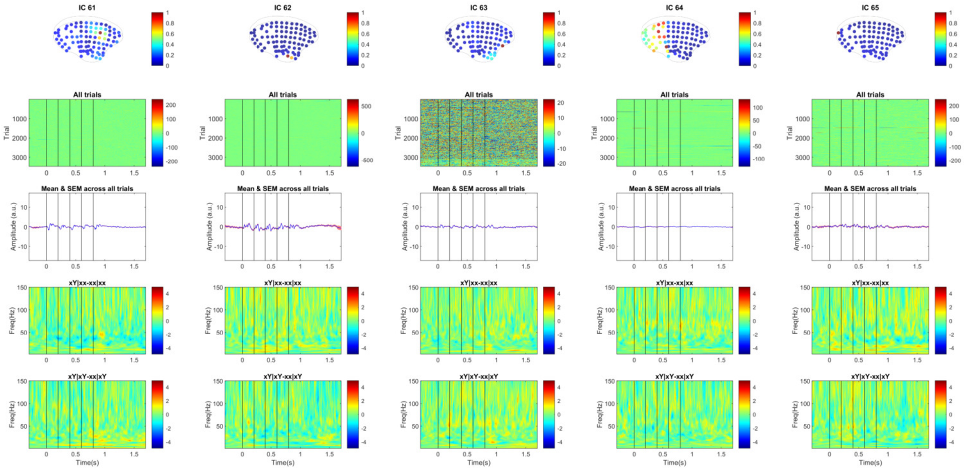

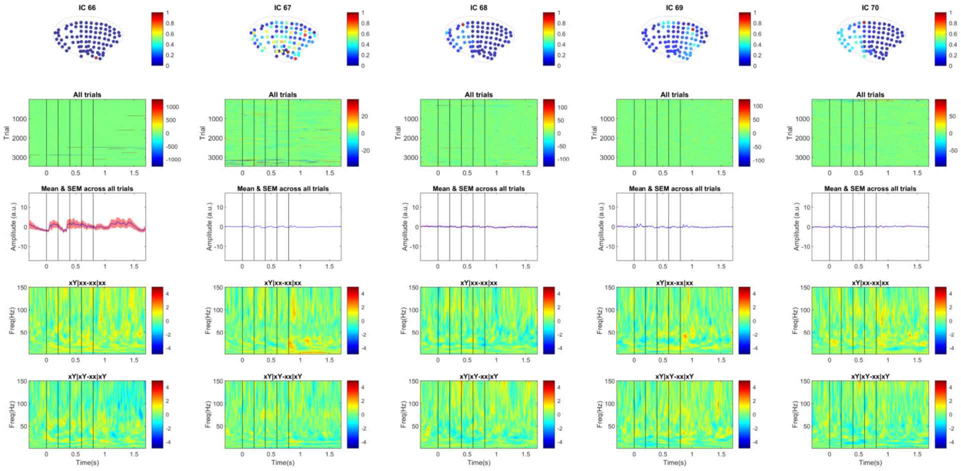

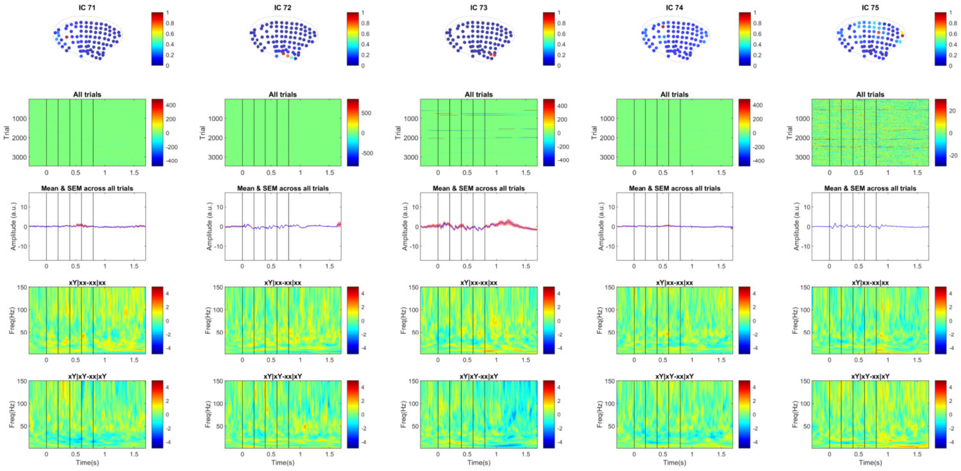

Subject: Yo (6/6)

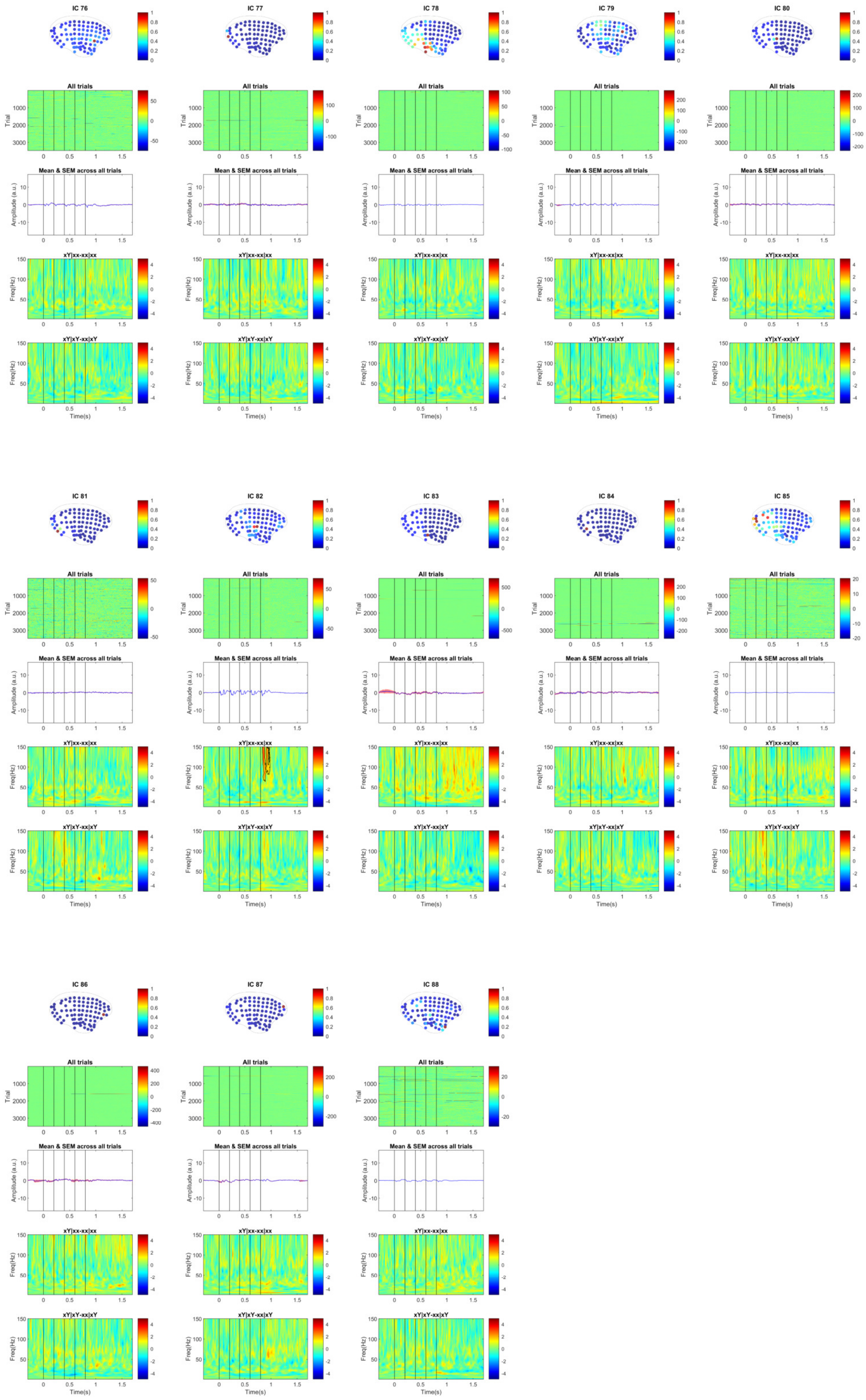

Subject: Ca (1/6)

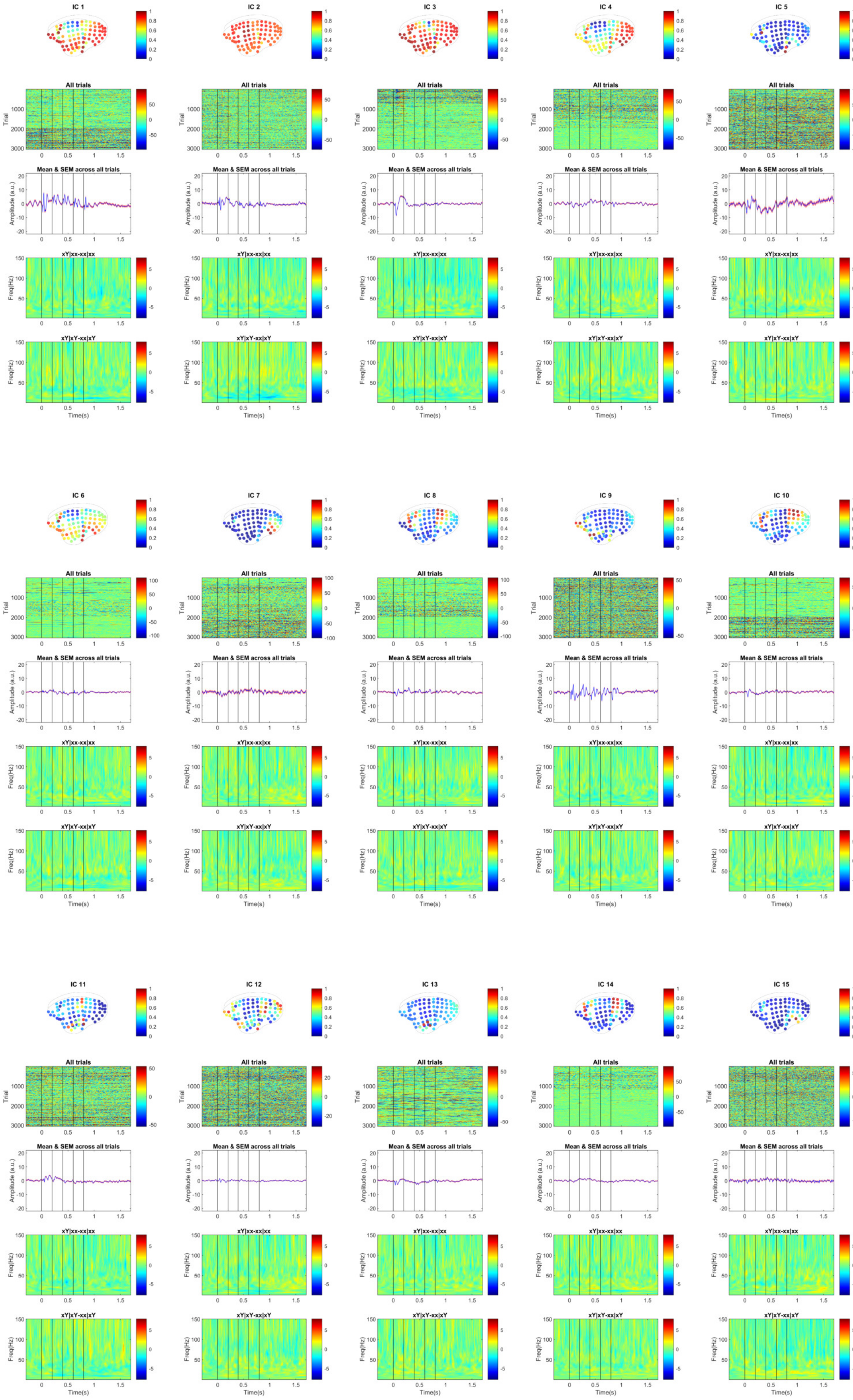

Subject: Ca (2/6)

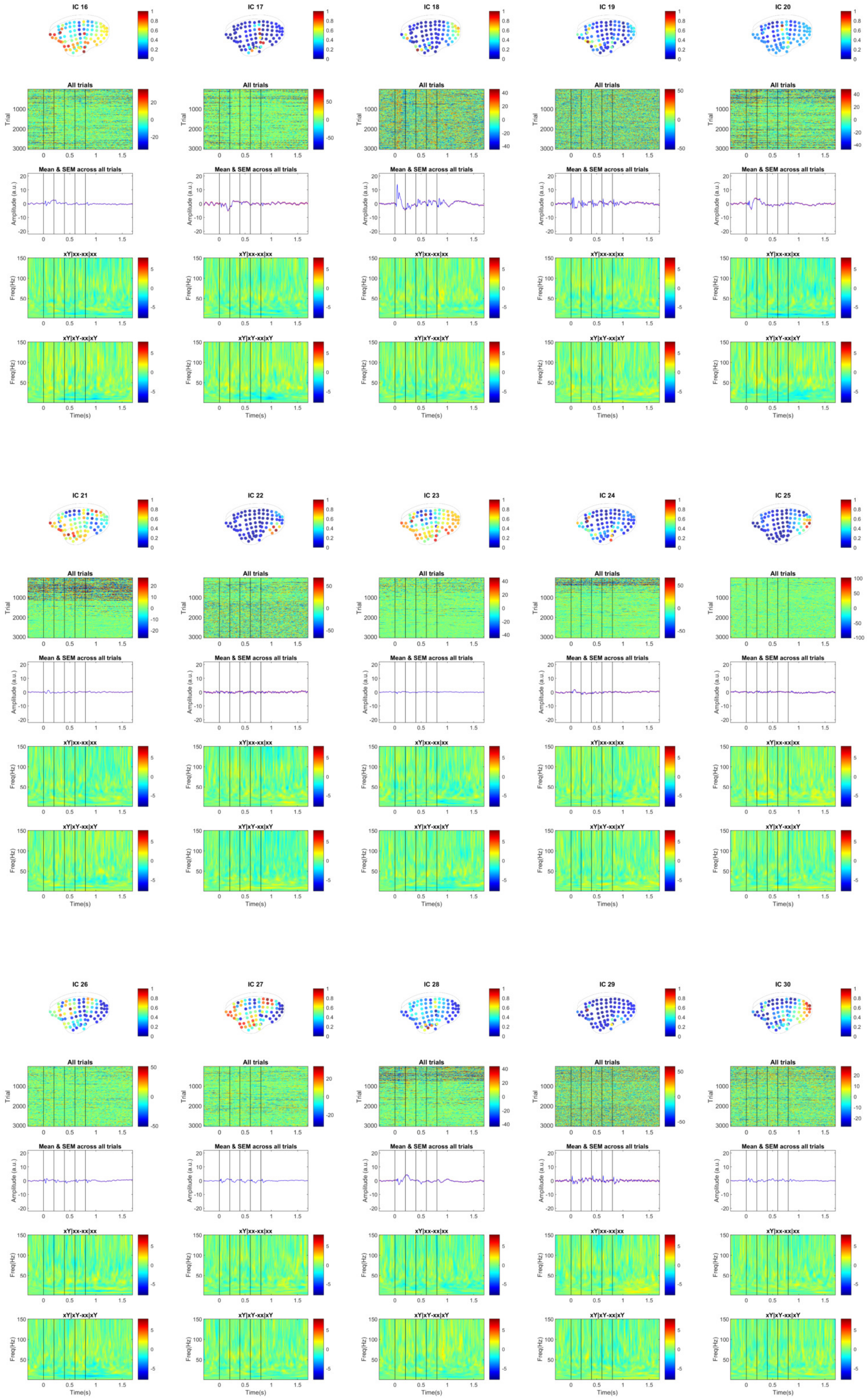

Subject: Ca (3/6)

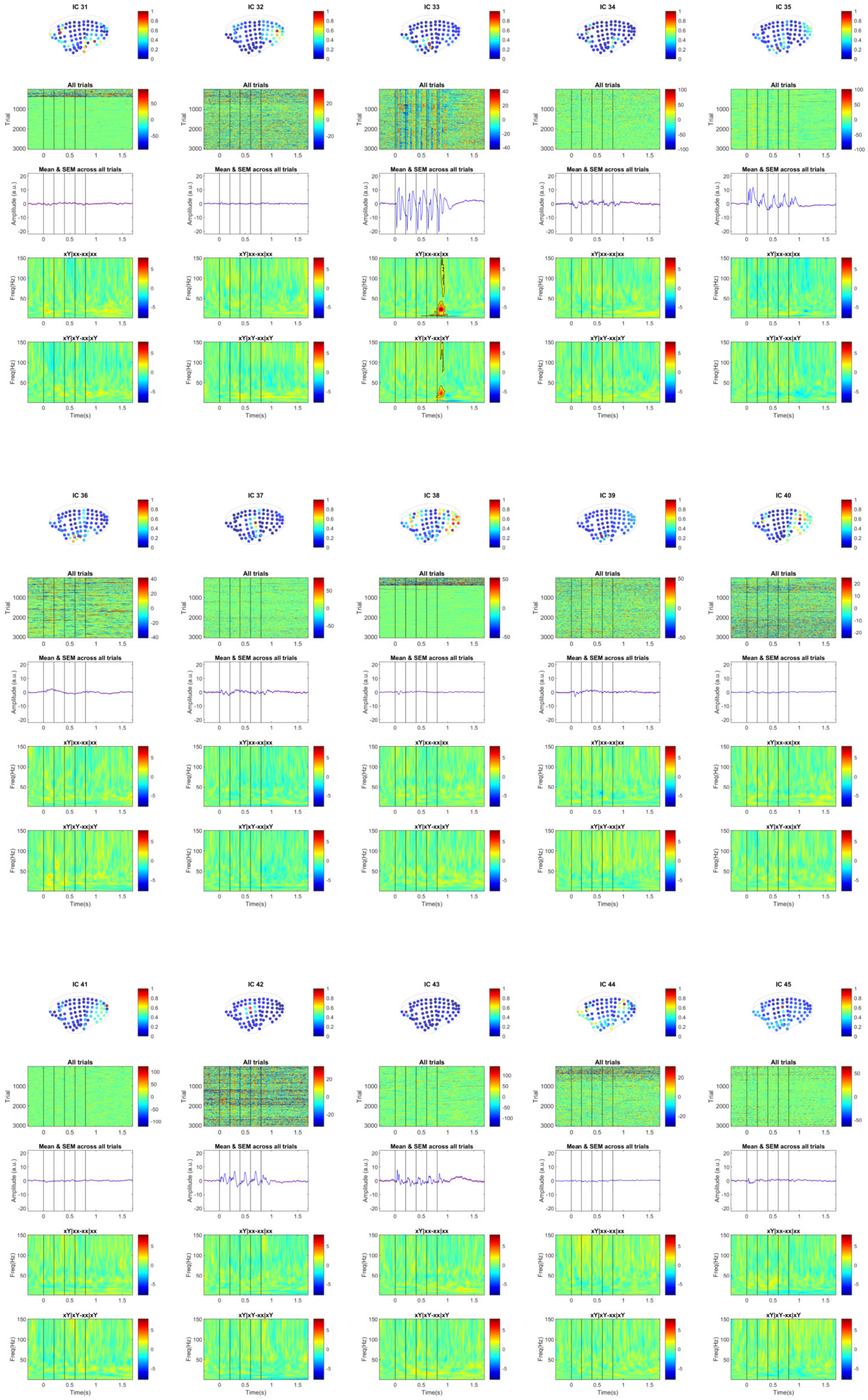

Subject: Ca (4/6)

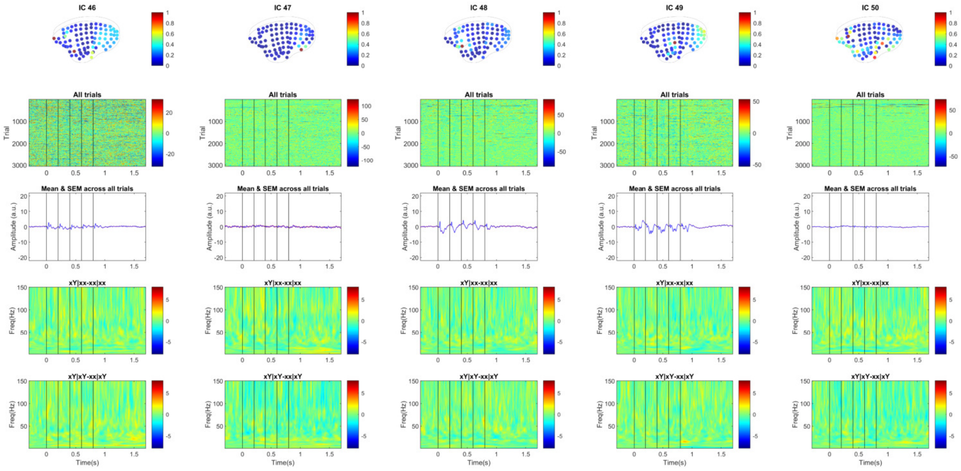

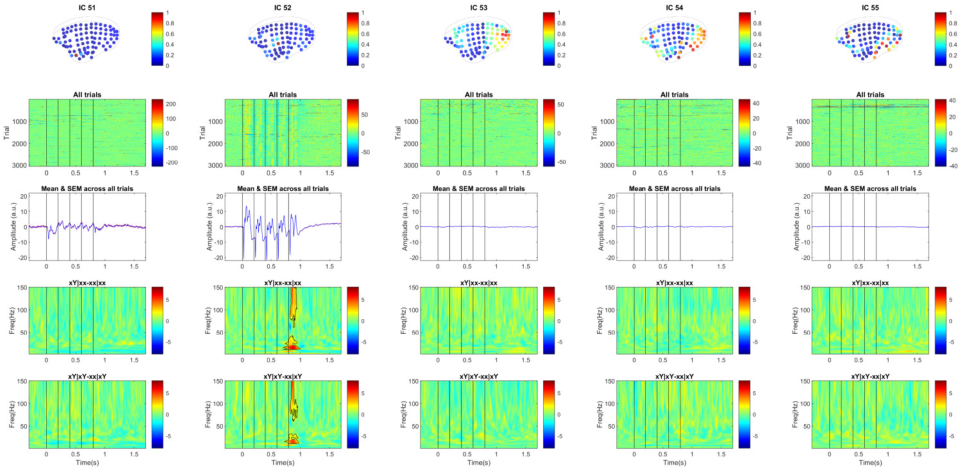

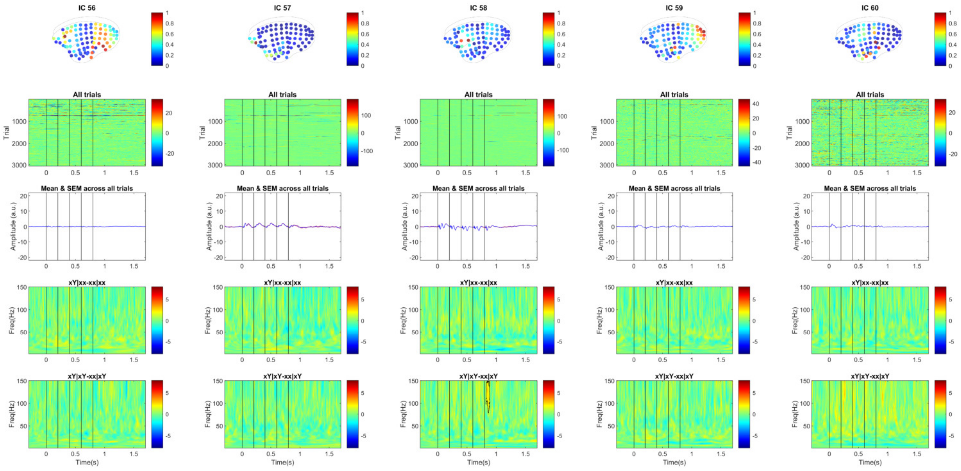

Subject: Ca (5/6)

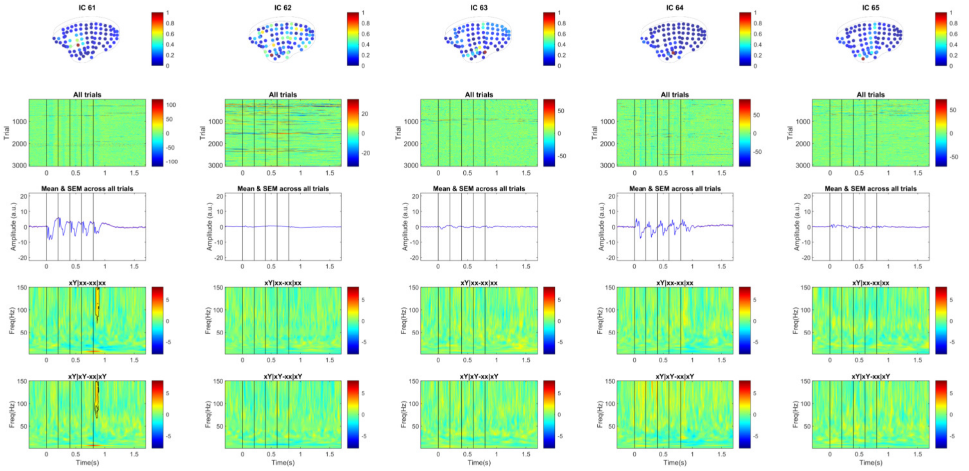

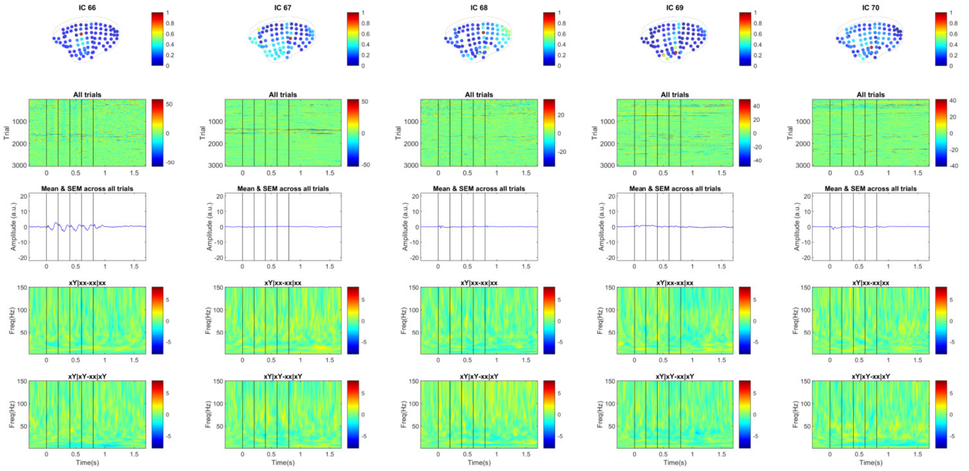

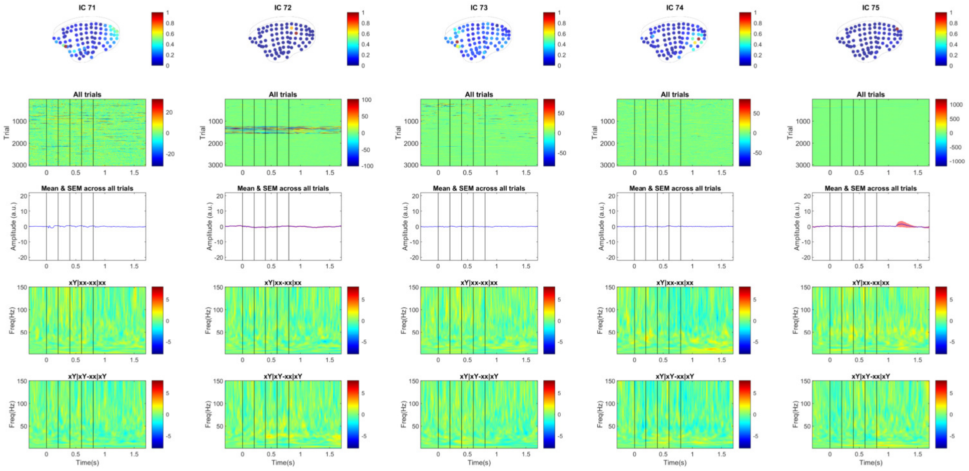

Subject: Ca (6/6)

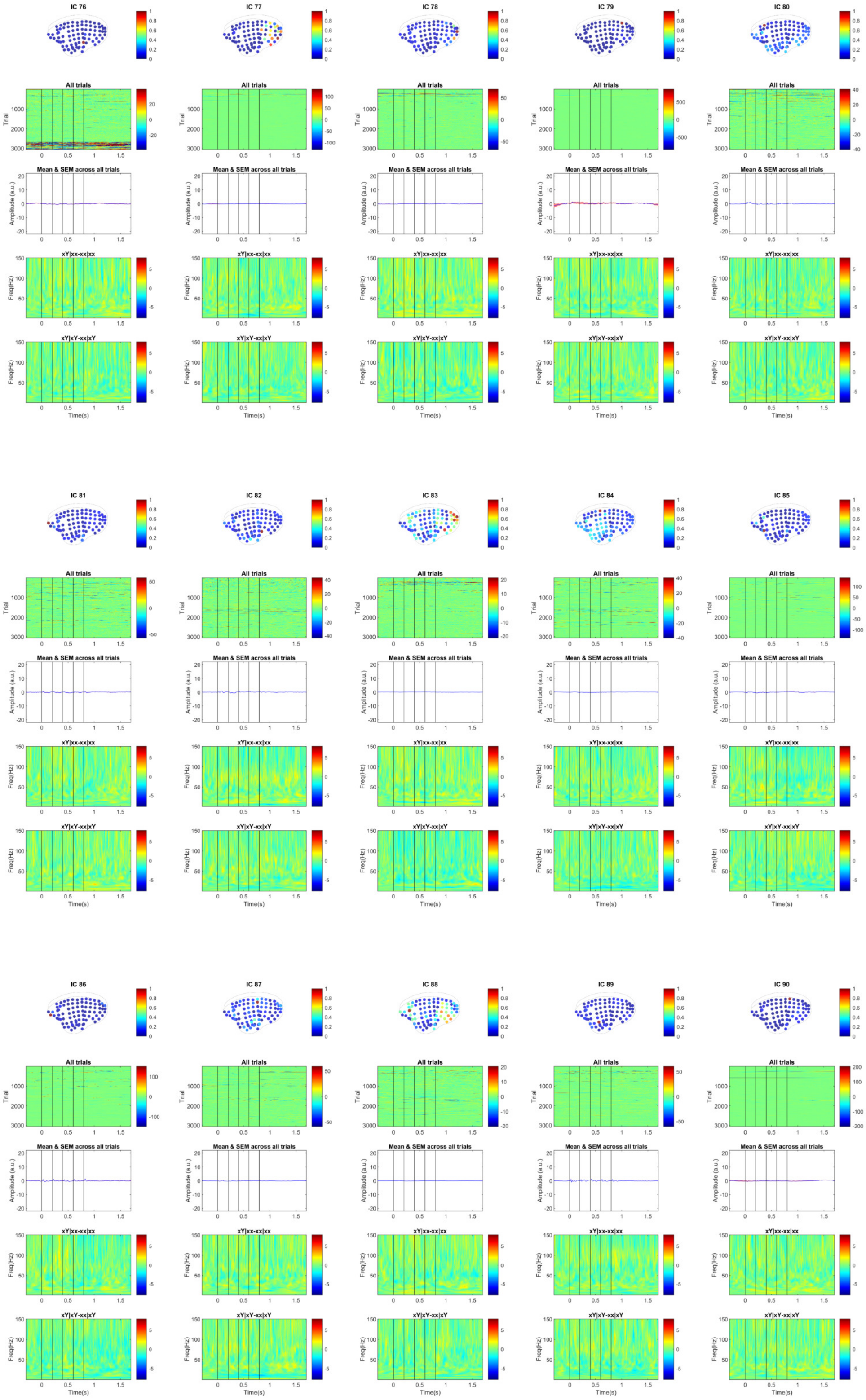

Subject: Rm (1/6)

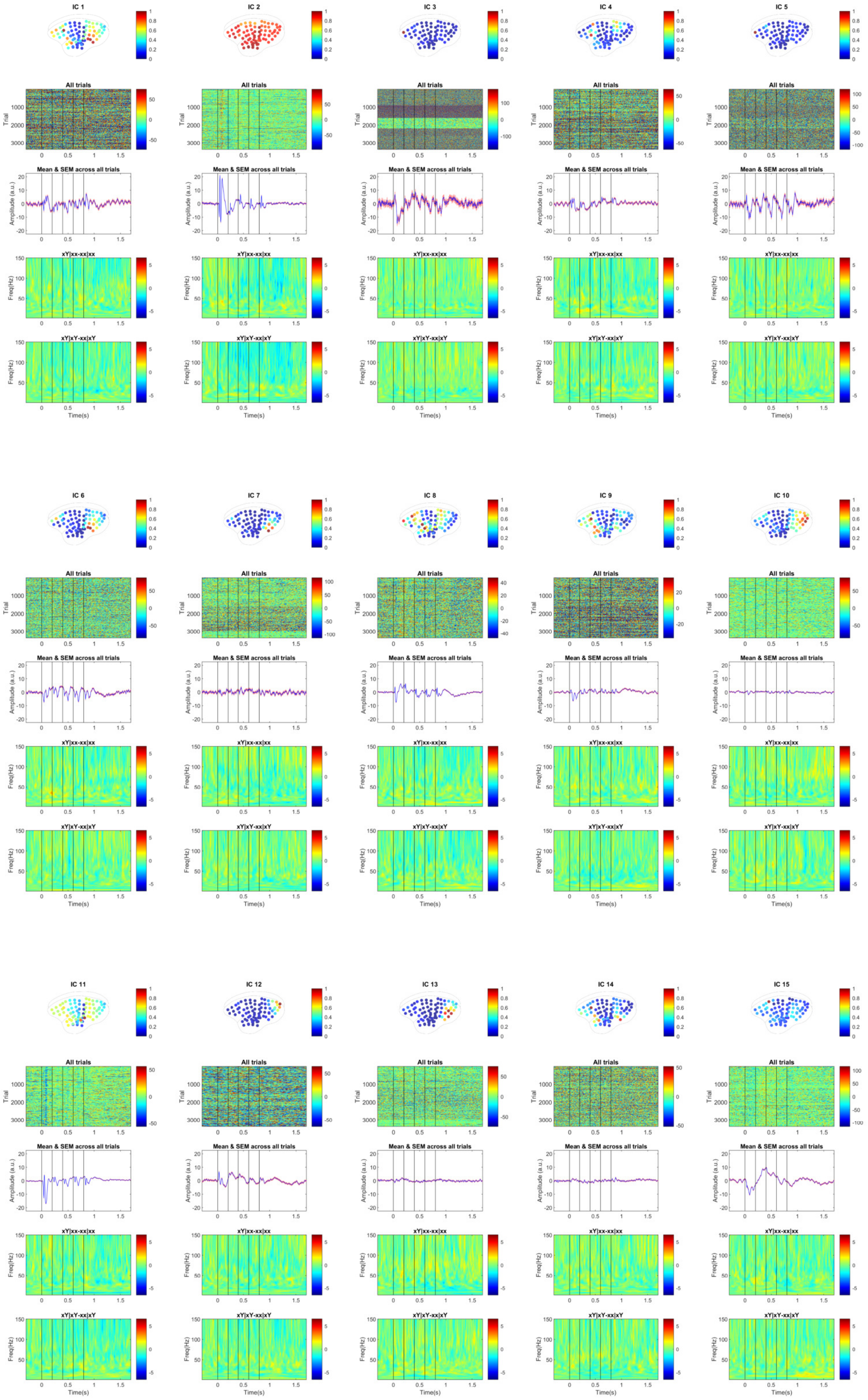

Subject: Rm (2/6)

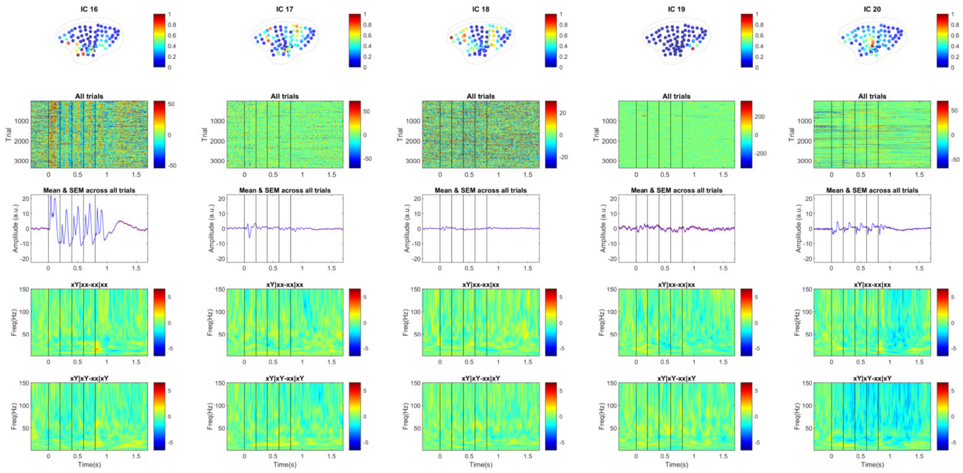

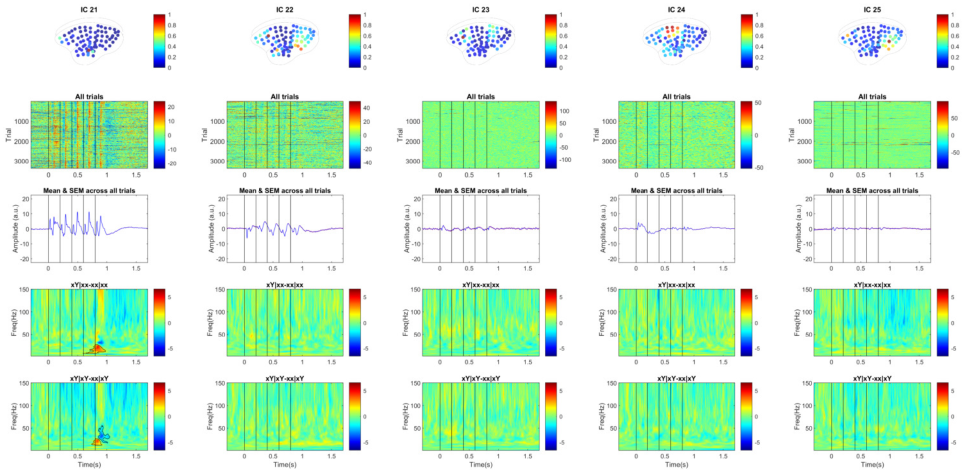

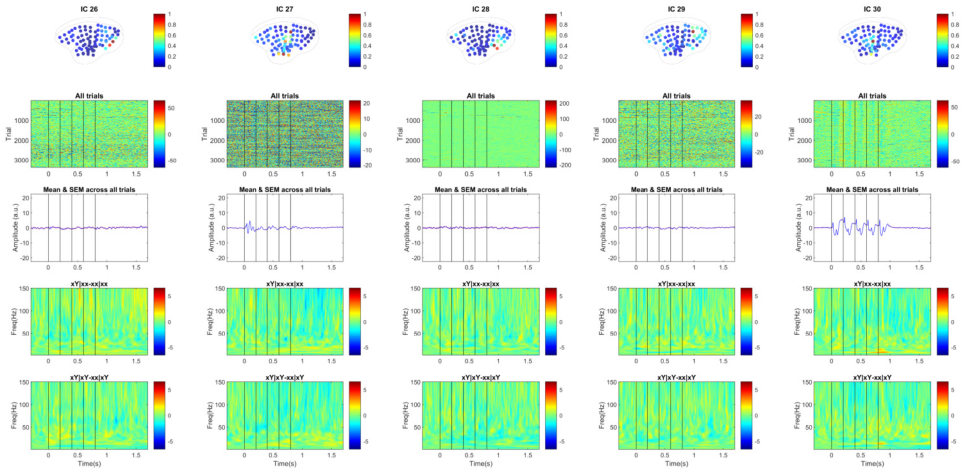

Subject: Rm (3/6)

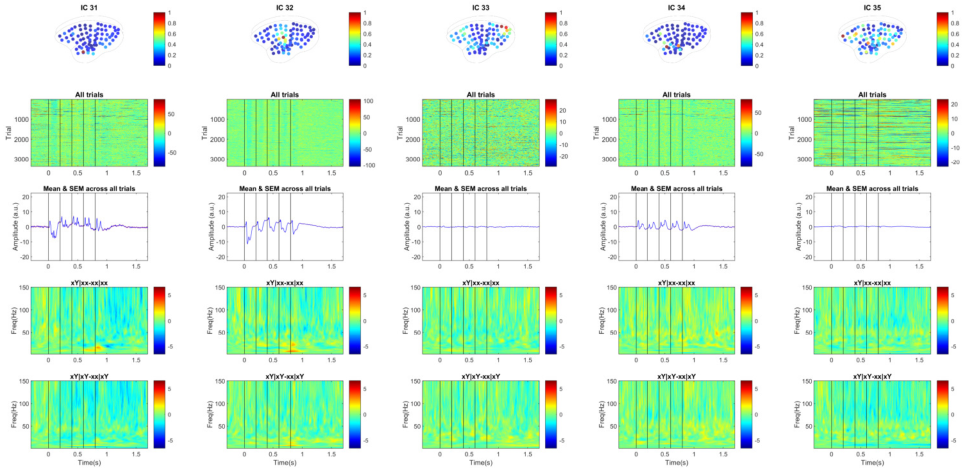

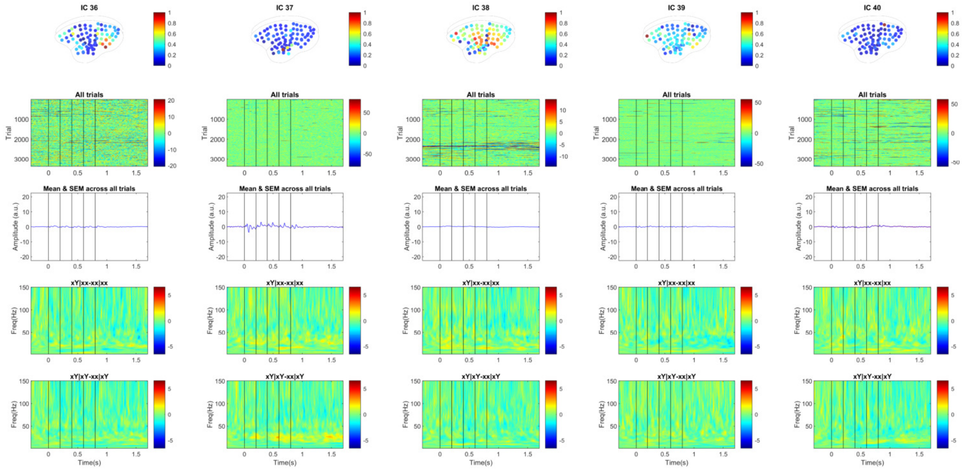

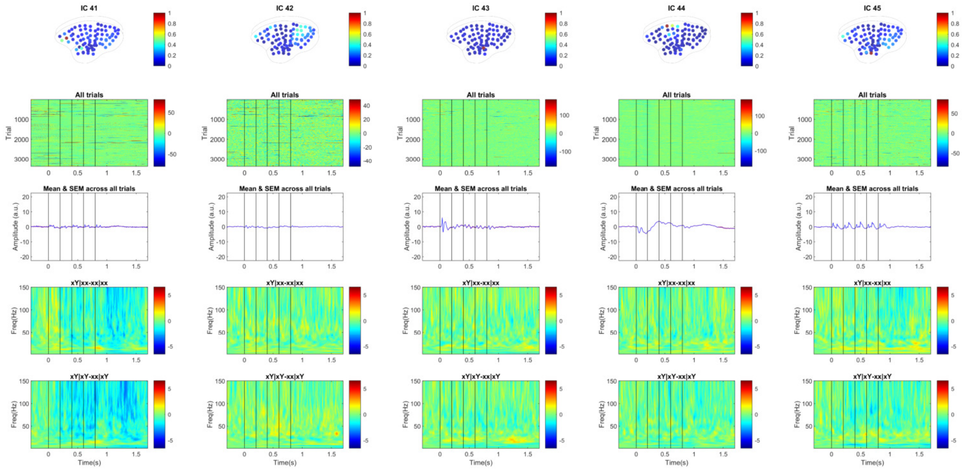

Subject: Rm (4/6)

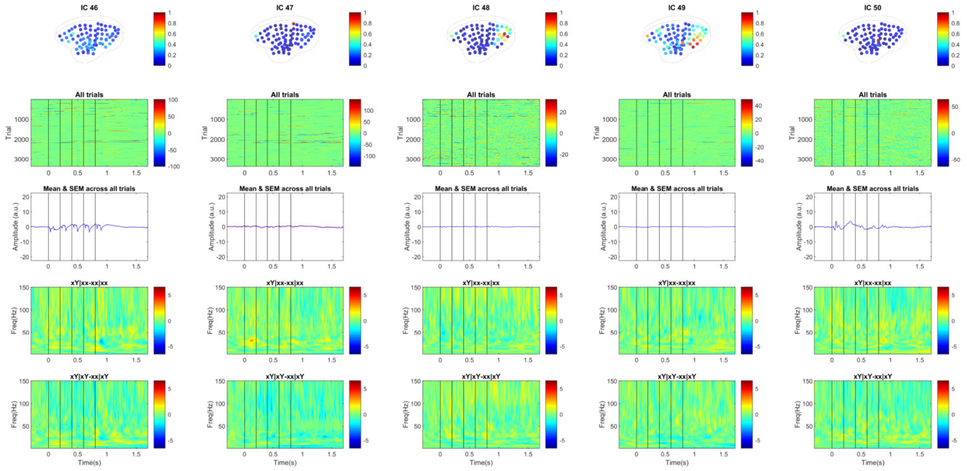

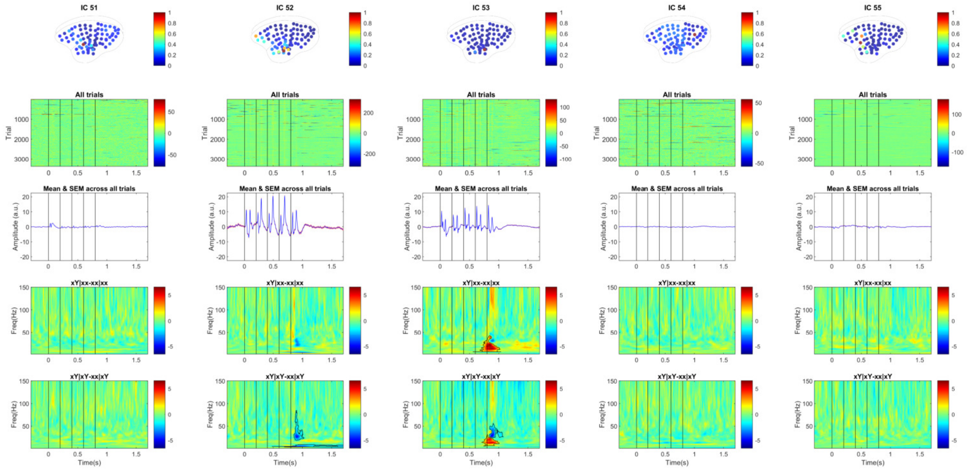

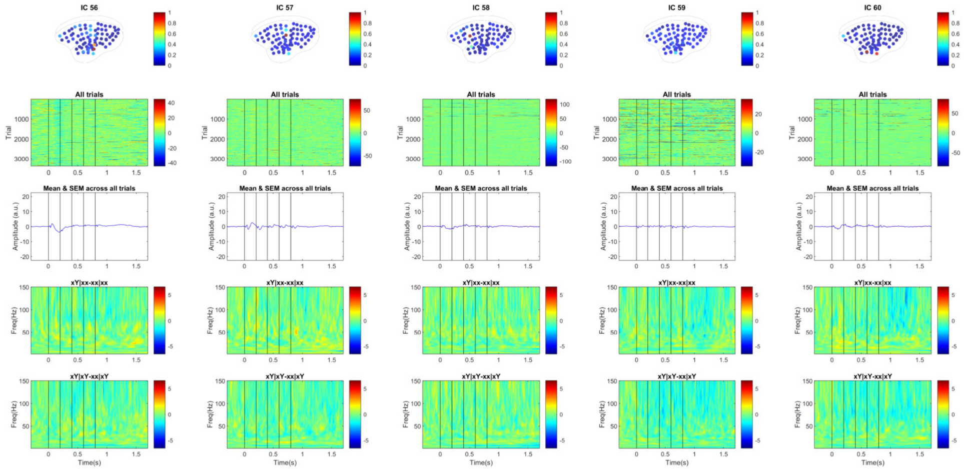

Subject: Rm (5/6)

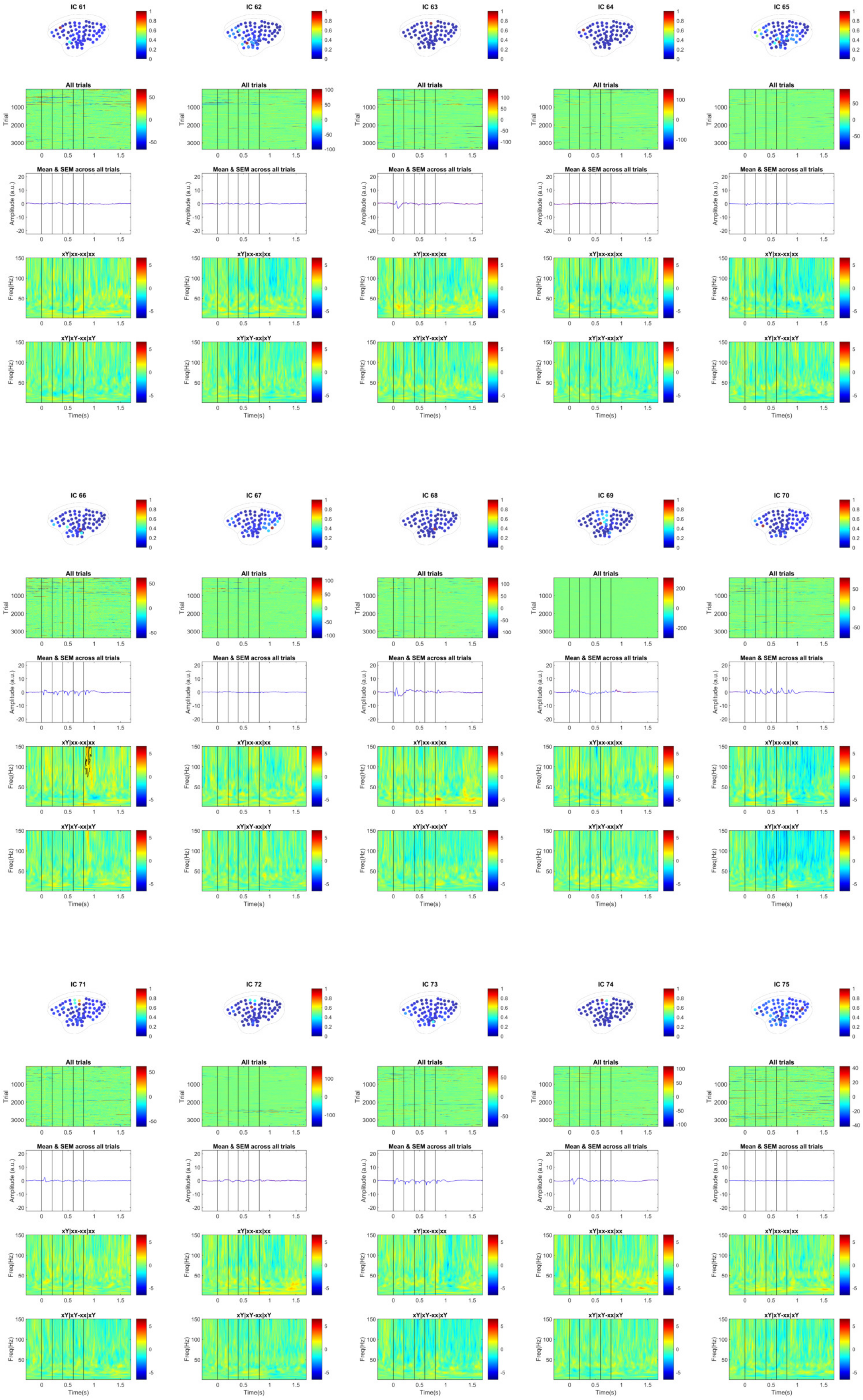

Subject: Rm (6/6)

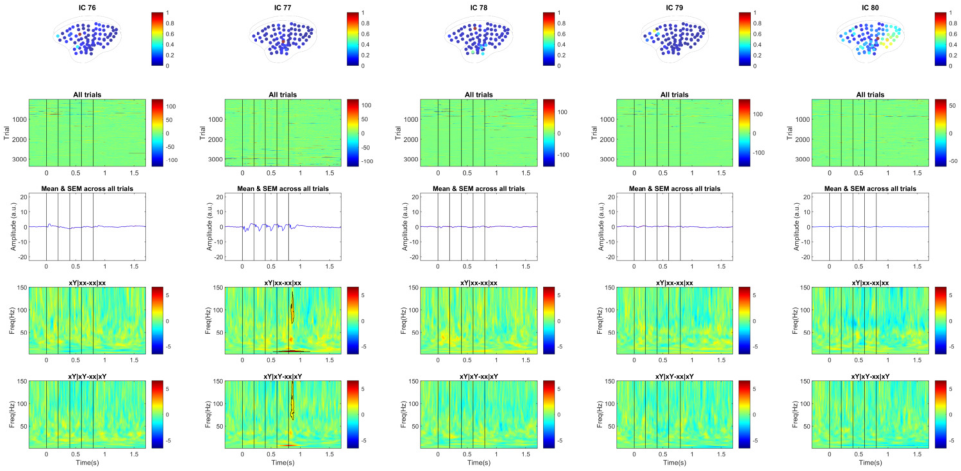

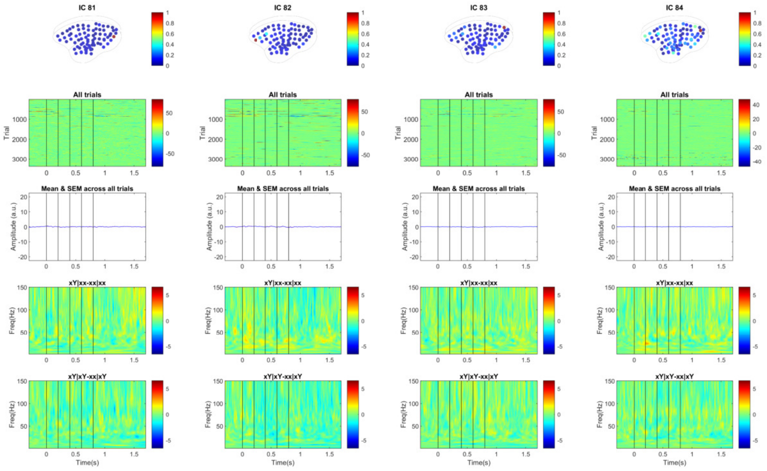

<u>Supplemental Figure S2</u>

Erroneous Predictive Coding Across Brain Hierarchies in a Non-Human Primate Model of Autism Spectrum Disorder

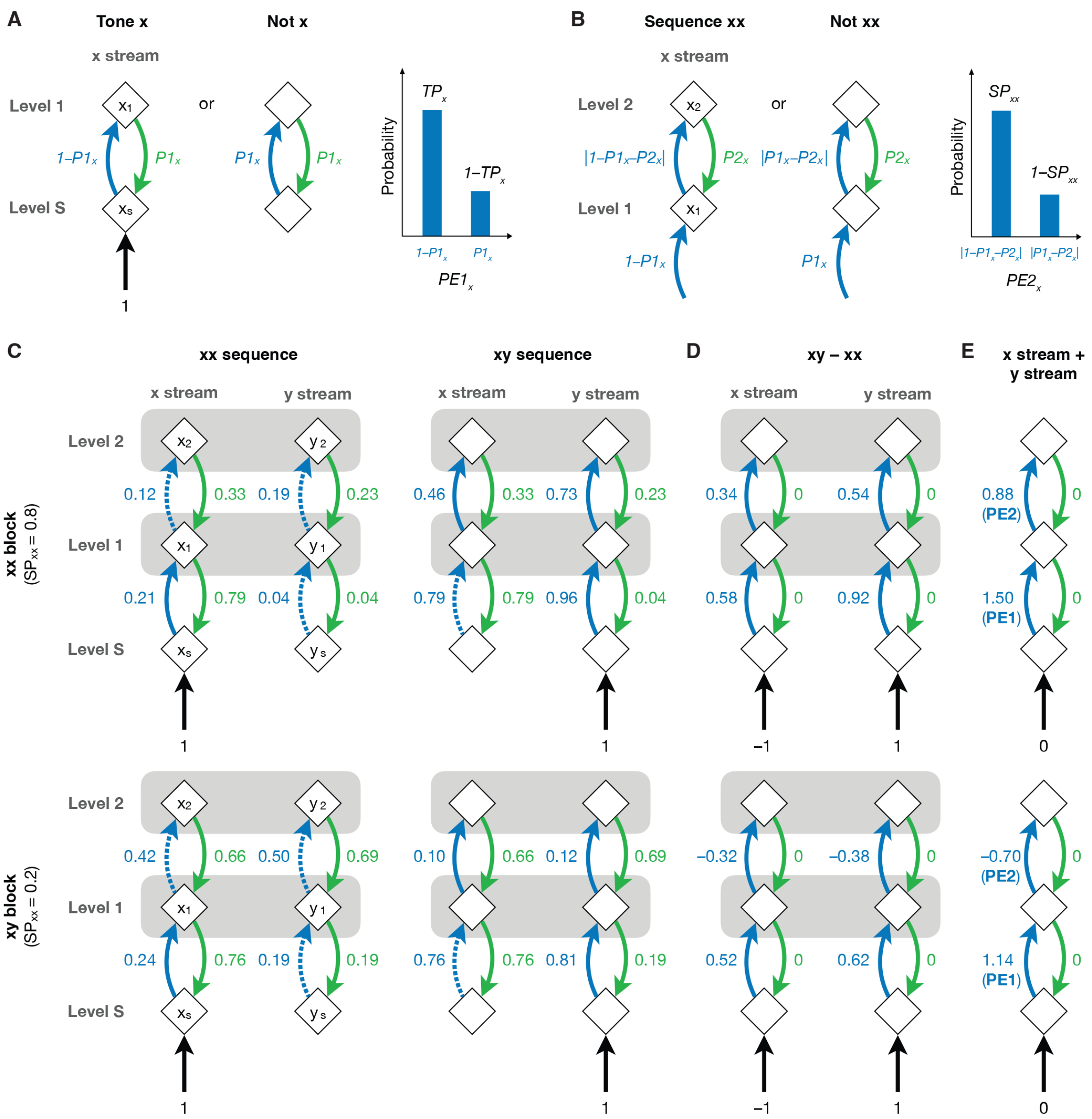

Figure S2. A Quantitative Predictive Coding Model. (A) The proposed neural operations in the x stream between Levels S and 1 during the presentation of tone x or not. An explanatory illustration of the probability distribution of the first-level prediction error in the x stream ($PE1_x$) is shown on the right. The neuronal populations (diamonds), prediction-error signal (blue arrow), prediction signal (green arrow), and sensory input (black arrow) are shown. (B) The neural operations in the x stream between Levels 1 and 2. An explanatory probability distribution of the second-level prediction error in the x stream ($PE2_x$) is shown on the right. (C) The complete model during the last tone in xx and xy sequences in the xx and xy blocks. The horizontal gray bars at Levels 1 and 2 indicate integration between the x and y streams

for computing transition and sequence probabilities, respectively. The negative errors, where the prediction is greater than the input or prediction error to be predicted, are shown in blue dashed arrows. (D) The contrast values (xy – xx) obtained from panel C. (E) The model values of PE1 and PE2 in the deviant responses by combining contrast values from the x and y streams in panel D.
