# Supplementary material for: Erroneous predictive coding across brain hierarchies in a non-human primate model of autism spectrum disorder
Source: Commun Biol. 2024 Jul 12;7:851. doi: 10.1038/s42003-024-06545-3 (PMC11239931; doi:10.1038/s42003-024-06545-3)
Supplement: Supplementary file 5 — Reporting summary [file 42003_2024_6545_MOESM5_ESM.pdf]

nature portfolio

Corresponding author(s): Zenas C. Chao

Last updated by author(s): 06/16/2024

# Reporting Summary

Nature Portfolio wishes to improve the reproducibility of the work that we publish. This form provides structure for consistency and transparency in reporting. For further information on Nature Portfolio policies, see our Editorial Policies and the Editorial Policy Checklist.

## Statistics

For all statistical analyses, confirm that the following items are present in the figure legend, table legend, main text, or Methods section.

| n/a | Confirmed | |
|---|---|---|
| ☐ | ☒ | The exact sample size (*n*) for each experimental group/condition, given as a discrete number and unit of measurement |
| ☒ | ☐ | A statement on whether measurements were taken from distinct samples or whether the same sample was measured repeatedly |
| ☐ | ☒ | The statistical test(s) used AND whether they are one- or two-sided<br>*Only common tests should be described solely by name; describe more complex techniques in the Methods section.* |
| ☒ | ☐ | A description of all covariates tested |
| ☐ | ☒ | A description of any assumptions or corrections, such as tests of normality and adjustment for multiple comparisons |
| ☐ | ☒ | A full description of the statistical parameters including central tendency (e.g. means) or other basic estimates (e.g. regression coefficient) AND variation (e.g. standard deviation) or associated estimates of uncertainty (e.g. confidence intervals) |
| ☐ | ☒ | For null hypothesis testing, the test statistic (e.g. *F*, *t*, *r*) with confidence intervals, effect sizes, degrees of freedom and *P* value noted<br>*Give P values as exact values whenever suitable.* |
| ☒ | ☐ | For Bayesian analysis, information on the choice of priors and Markov chain Monte Carlo settings |
| ☒ | ☐ | For hierarchical and complex designs, identification of the appropriate level for tests and full reporting of outcomes |
| ☒ | ☐ | Estimates of effect sizes (e.g. Cohen's *d*, Pearson's *r*), indicating how they were calculated |

*Our web collection on statistics for biologists contains articles on many of the points above.*

## Software and code

Policy information about availability of computer code

| | |
|---|---|
| Data collection | All experimental protocols were programmed with the MATLAB-based PsychotoolBox. |
| Data analysis | EEG analysis was done by using MATLAB toolboxes: EEGLAB, FieldTrip, and N-way toolbox (details provided in Methods). MATLAB code for model computation is available in Zenodo (https://doi.org/10.5281/zenodo.7084568). |

For manuscripts utilizing custom algorithms or software that are central to the research but not yet described in published literature, software must be made available to editors and reviewers. We strongly encourage code deposition in a community repository (e.g. GitHub). See the Nature Portfolio guidelines for submitting code & software for further information.

## Data

Policy information about availability of data

All manuscripts must include a data availability statement. This statement should provide the following information, where applicable:

- Accession codes, unique identifiers, or web links for publicly available datasets
- A description of any restrictions on data availability
- For clinical datasets or third party data, please ensure that the statement adheres to our policy

The ECoG data and source data underlying the figures are available from the corresponding author on reasonable request.

## Human research participants

Policy information about studies involving human research participants and Sex and Gender in Research.

| | |
|---|---|
| Reporting on sex and gender | *Use the terms sex (biological attribute) and gender (shaped by social and cultural circumstances) carefully in order to avoid confusing both terms. Indicate if findings apply to only one sex or gender; describe whether sex and gender were considered in study design whether sex and/or gender was determined based on self-reporting or assigned and methods used. Provide in the source data disaggregated sex and gender data where this information has been collected, and consent has been obtained for sharing of individual-level data; provide overall numbers in this Reporting Summary. Please state if this information has not been collected. Report sex- and gender-based analyses where performed, justify reasons for lack of sex- and gender-based analysis.* |
| Population characteristics | *Describe the covariate-relevant population characteristics of the human research participants (e.g. age, genotypic information, past and current diagnosis and treatment categories). If you filled out the behavioural & social sciences study design questions and have nothing to add here, write "See above."* |
| Recruitment | *Describe how participants were recruited. Outline any potential self-selection bias or other biases that may be present and how these are likely to impact results.* |
| Ethics oversight | *Identify the organization(s) that approved the study protocol.* |

Note that full information on the approval of the study protocol must also be provided in the manuscript.

# Field-specific reporting

Please select the one below that is the best fit for your research. If you are not sure, read the appropriate sections before making your selection.

☒ Life sciences ☐ Behavioural & social sciences ☐ Ecological, evolutionary & environmental sciences

For a reference copy of the document with all sections, see nature.com/documents/nr-reporting-summary-flat.pdf

# Life sciences study design

All studies must disclose on these points even when the disclosure is negative.

| | |
|---|---|
| Sample size | In this study, we utilized three healthy animals alongside two animals treated with valproic acid (VPA). The availability of VPA-treated animals was limited, attributable to the intricate process required for their production. |
| Data exclusions | No data from any subject was excluded. For one animal, 8 electrodes were removed during the implantation due to tissue adhesions. For data preprocessing, bad channels were then removed by visual inspections: channels 6, 7, 8, 23 and 80 were removed in Rc; channels 8, 68, 71, 81, 83, 85, 87, and 88 were removed from Yo; channels 2, 44, 48, 61, 63, 64 were removed in Ca, channels 1, 6, 7, 8, 9, 10, 43, 43, 49, 25 and 81 were removed in Rm. No trials were removed from the analysis. All these information are detailed in the manuscript. |
| Replication | The reproducibility of the current results were verified by rigorous statistical testing (details in Methods). |
| Randomization | Randomization is not relevant to our study. |
| Blinding | The data collected from the five animals were analyzed blindly, without prior knowledge of which animals had been treated with VPA. |

# Reporting for specific materials, systems and methods

We require information from authors about some types of materials, experimental systems and methods used in many studies. Here, indicate whether each material, system or method listed is relevant to your study. If you are not sure if a list item applies to your research, read the appropriate section before selecting a response.


March 2021

## Materials & experimental systems

| n/a | Involved in the study |
| --- | --- |
| ☒ | ☐ Antibodies |
| ☒ | ☐ Eukaryotic cell lines |
| ☒ | ☐ Palaeontology and archaeology |
| ☐ | ☒ Animals and other organisms |
| ☒ | ☐ Clinical data |
| ☒ | ☐ Dual use research of concern |

## Methods

| n/a | Involved in the study |
| --- | --- |
| ☒ | ☐ ChIP-seq |
| ☒ | ☐ Flow cytometry |
| ☒ | ☐ MRI-based neuroimaging |

# Animals and other research organisms

Policy information about studies involving animals; ARRIVE guidelines recommended for reporting animal research, and Sex and Gender in Research

| | |
| --- | --- |
| Laboratory animals | We used five adult common marmosets (Callithrix jacchus; three males and two females, 320–450 g, 22–42 months). |
| Wild animals | The study did not involve wild animals. |
| Reporting on sex | In our study, we included only two female animals—one healthy and one treated with VPA—and did not take sex into account in our analysis. |
| Field-collected samples | The study did not involve collecting field samples. |
| Ethics oversight | All procedures of the ECoG study at RIKEN were conducted in accordance with a protocol approved by the RIKEN Ethical Committee. All procedures of the VPA preparation and ECoG study at NCNP were conducted in accordance with NIH guidelines and the "Guide for the Care and Use of Primate Laboratory Animals" published by the National Institute of Neurological Research, National Center of Neurology and Psychiatry, and approved by the Animal Research Committee of NCNP. |

Note that full information on the approval of the study protocol must also be provided in the manuscript.
